# Mitigating candidiasis with acarbose by targeting *Candida albicans* α-glucosidase: in-silico, in-vitro and transcriptomic approaches

**DOI:** 10.1038/s41598-024-62684-x

**Published:** 2024-05-24

**Authors:** Helma David, Sahana Vasudevan, Adline Princy Solomon

**Affiliations:** 1 Quorum Sensing Laboratory, Centre for Research in Infectious Diseases (CRID), School of Chemical and Biotechnology, SASTRA Deemed to be University, Thanjavur, 613401 India; 2 Institute for Stem Cell Science and Regenerative Medicine (inStem), Bangalore, 560065 India

**Keywords:** *Candida albicans*, Candidiasis, α-glucosidase, Acarbose, Glycomimetics, Mannoproteins, Drug repurposing, Drug discovery, Microbiology, Biofilms, Fungi, Pathogens

## Abstract

Biofilm-associated candidiasis poses a significant challenge in clinical settings due to the limited effectiveness of existing antifungal treatments. The challenges include increased pathogen virulence, multi-drug resistance, and inadequate penetration of antimicrobials into biofilm structures. One potential solution to this problem involves the development of novel drugs that can modulate fungal virulence and biofilm formation, which is essential for pathogenesis. Resistance in *Candida albicans* is initiated by morphological changes from yeast to hyphal form. This transition triggers a series of events such as cell wall elongation, increased adhesion, invasion of host tissues, pathogenicity, biofilm formation, and the initiation of an immune response. The cell wall is a critical interface for interactions with host cells, primarily through various cell wall proteins, particularly mannoproteins. Thus, cell wall proteins and enzymes are considered potential antifungal targets. In this regard, we explored α-glucosidase as our potential target which plays a crucial role in processing mannoproteins. Previous studies have shown that inhibition of α-glucosidase leads to defects in cell wall integrity, reduced adhesion, diminished secretion of hydrolytic enzymes, alterations in immune recognition, and reduced pathogenicity. Since α-glucosidase, primarily converts carbohydrates, our study focuses on FDA-approved carbohydrate mimic drugs (Glycomimetics) with well-documented applications in various biological contexts. Through virtual screening of 114 FDA-approved carbohydrate-based drugs, a pseudo-sugar Acarbose, emerged as a top hit. Acarbose is known for its pharmacological potential in managing type 2 diabetes mellitus by targeting α-glucosidase. Our preliminary investigations indicate that Acarbose effectively inhibits *C. albicans* biofilm formation, reduces virulence, impairs morphological switching, and hinders the adhesion and invasion of host cells, all at very low concentrations in the nanomolar range. Furthermore, transcriptomic analysis reveals the mechanism of action of Acarbose, highlighting its role in targeting α-glucosidase.

## Introduction

Fungal infections caused by *C. albicans* are a significant global health concern, accounting for approximately 70% of all fungal infections. These infections are associated with a high fatality rate, ranging from 30 to 60%^1^. *C. albicans* can manifest infections in the various parts of the body and can progress to life-threatening systemic infections^2,3^. The recurrence of candida infections of the skin and mucosal surfaces is identified as chronic mucocutaneous candidiasis (CMC) and mainly occurs in individuals with primary or acquired immunodeficiencies^4^. In addition, the World Health Organisation recently identified *C. albicans* as a critical priority pathogen in 2022^5^. The connection between candidiasis and various systemic illnesses, such as diabetes, cancer, and autoimmune disorders, underscores the far-reaching implications of these infections^6^. While *C. albicans* is normally present in several parts of the human body as a commensal organism, with its repertoire of virulence factors, it can evolve rapidly into a life-threatening pathogen^7^.

The existing arsenal of antifungal drugs is notably limited, primarily comprising four classes: polyenes, azoles, pyrimidine analogues, and echinocandins^3^. Moreover, even the more recently developed antifungals, such as echinocandins, have encountered resistance in several *Candida* species^8^. *C. albicans* has evolved intricate mechanisms to resist current antifungal treatments, as these drugs target crucial metabolic pathways within the organism, leading to either inherent or acquired resistance^9^. Therefore, it is essential to explore novel approaches, focusing on molecular targets related to the pathogenesis and virulence of *C. albicans*. The most notable hallmarks of *C. albicans* pathogenesis are filamentation and biofilm formation. While the filamentous form helps in evading the immune system and invading the host tissue, biofilm formation enhances the antifungal resistance to an alarmingly increasing fold^3^. Thus, novel therapies that target biofilm formation and filamentation inhibition should be harnessed.

Over the past decades, an extensive understanding of the virulence mechanisms of *C. albicans* has resulted in the identification of several specific targets and pathways for the development of potential antifungal and anti-virulence drugs^10^. The cell wall of *C. albicans*, being the primary point of contact between the fungus and its host, plays a pivotal role in their interaction. Also, cell wall components are explored as promising antifungal targets^11^. The cell wall of *C. albicans* is augmented with highly glycosylated mannoproteins in the outer layer and is bound to the β-glucan/chitin inner layer through the lateral chains of β-1,6-glucan or, to a lesser extent, to β1,3-glucan^12^. Mannoproteins, which are processed by alpha-glycosidase, play pivotal roles in adhesion, antigenicity, controlling the host immune response, and innate immune cell’s ability to recognize this fungus^13^. Alpha-glucosidase I and II in the endoplasmic reticulum are encoded by the *CWH41* gene and *ROT2* gene, respectively^14^. Ora et al. previously showed the significance of α-glucosidase as an enzyme for cell wall synthesis in *C. albicans* and the detrimental effects when cells lacked alpha-glucosidase. *cwh41Δ* and *rot2Δ* null mutants displayed delayed filamentation and shorter germ tubes, reduced contents of mannan in the cell wall, and decreased virulence in the murine model infected, demonstrating that α-glucosidases are vital for *C. albicans* growth and virulence^14^. Therefore, novel antifungal compounds against alpha-glucosidases have a promising future as potential drugs.

The drug repurposing approach is an emerging approach to identifying new pharmacological indications of already existing or failed FDA-approved or investigational drugs. Given the time-consuming, resource-intensive, and expensive nature of traditional drug discovery, this novel method holds promise as a more efficient alternative in reducing the risk of failure. It has gained attention as a potential strategy to combat infectious diseases due to its advantages, as it resolves the bottlenecks associated with modern drug development such as the lack of new chemical entity (NCE), exorbitant costs, time consumption, and the need for extensive toxicity testing^15^. Among the various drug classes investigated in drug repurposing approaches, glycomimetic drugs emerge as a pivotal category. Glycomimetics have emerged as promising candidates for drug development due to their unique ability to mimic the structural and functional aspects of carbohydrates. Carbohydrates play crucial roles in various biological processes, and their mimetics offer a synthetic approach to modulate these processes for therapeutic purposes. By replicating the bioactive functions of carbohydrates, glycomimetics become valuable tools for influencing cell adhesion, signalling, and recognition. The poly-pharmacological potential of glycomimetic drugs seamlessly aligns with the intricate nature of drug repurposing strategies, facilitating simultaneous targeting of multiple pathways, and thereby enhancing the comprehensiveness of therapeutic interventions^16–18^.

Based on this premise we aim to develop a next-generation antifungal targeting *C. albicans* α-glucosidase by multimodal strategies using in silico, in vitro approaches and prove its mechanism of action through transcriptomic studies.

## Results and discussion

### Screening of FDA-approved glycomimetic drugs with α-glucosidase

#### Model building and validation

**Figure 1:**
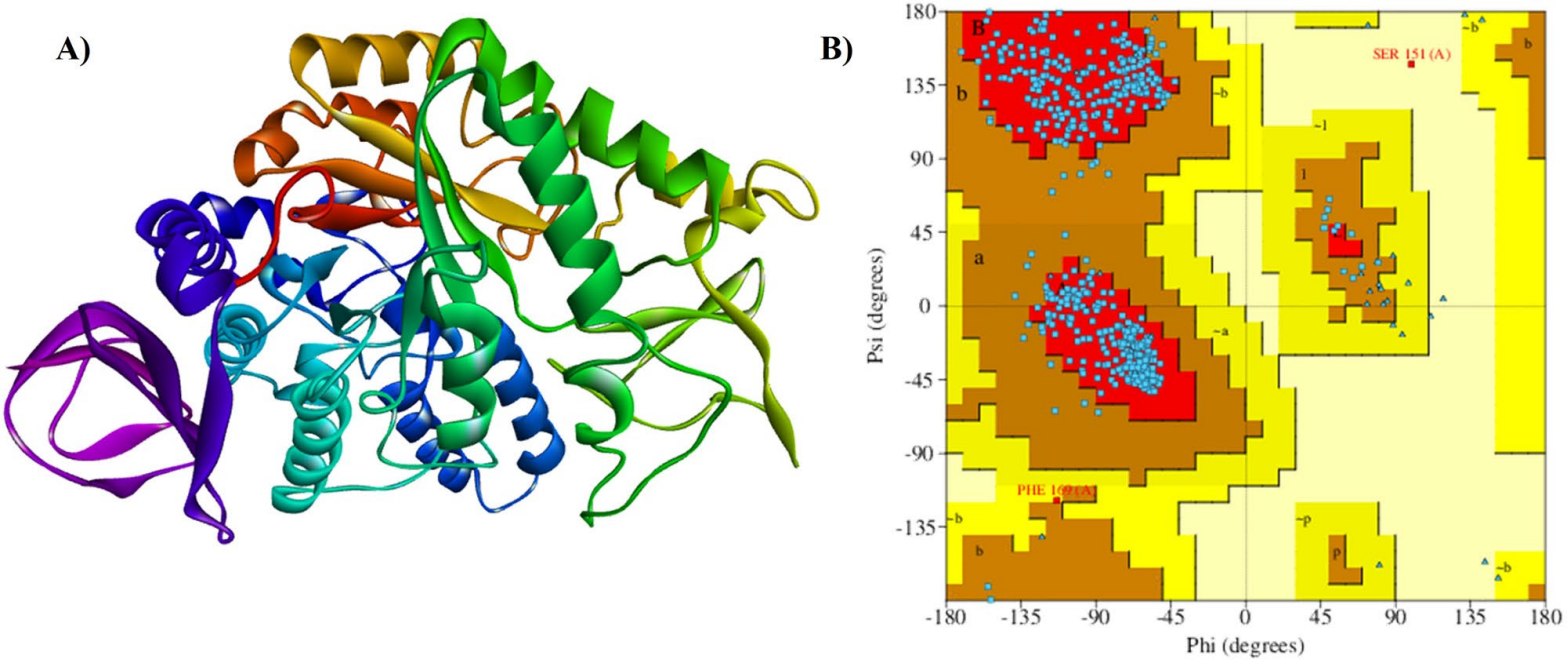
(**A**) Structural Representation and of Protein QSL-M1 Modelled by Alphafold2 (**B**) Structure validation of the homology modelled α-glucosidase protein by Ramachandran Plot using the PROCHECK.

In the realm of structural protein design and functional investigation, homology modeling stands out as a prominent method. This approach involves predicting the three-dimensional structure of a target protein sequence by aligning it with a known protein template, enabling a comprehensive understanding of protein structures and functions^19^. Alphafold2, an advanced protein structure prediction method, leverages deep learning algorithms and evolutionary conservation principles^20^. Alphafold2 generated five distinct models (QSL-M1, QSL-M2, QSL-M3, QSL-M4, and QSL-M5 (Table S1)) through an end-to-end deep neural network, meticulously trained to predict protein structures from amino acid sequences, incorporating data from homologous proteins and multiple sequence alignments to enhance predictive accuracy. The model quality is assessed using Erratplot, and subsequent refinement is carried out with GalaxyRefine. GalaxyRefine iteratively rebuilds side-chain conformations, starting from the protein core and extending outward to address steric clashes by using higher probability rotamers as needed. It also evaluates the interactions of neighbouring Cβ atoms around each side chain and reverts to the expected canonical conformation if deviations are observed in relation to the amino acid’s surface exposure level^21^. After refinement, the Erratplot quality score is re-evaluated, and QSL-M1 exhibits a quality score of 93.9 (Fig. 1A). Additionally, a 100% conservation of amino acid residues is observed between the Multiple Sequence Alignment of α-glucosidase and QSL-M1. Furthermore, the model undergoes analysis through a Ramachandran Plot using Procheck, revealing that 95.5% of the residues fall within the allowed regions and only 0.2% in the disallowed regions (Fig. 1B). The AlphaFold2-generated model demonstrates a high level of accuracy, leading to the selection of QSL-M1 for subsequent docking studies.

#### Molecular docking

The QSL-M1 is prepared using the protein preparation wizard (PrepWizard) by Schrodinger. The active site catalytic residues collected from scientific literature were confirmed using CastP for QSL-M1. Three residues, ASP206, GLU263, and ASP338, were observed to be inside the binding pocket of QSL-M1 ASP206 is the catalytic nucleophile involved in the hydrolysis of terminal, non-reducing (1- > 4)-linked alpha-D-glucose residues with the alpha-D-glucose release. GLU263 is the proton donor and ASP338 is the transition state stabilizer. The grid box is fixed around these three conserved residues for docking.

**Figure 2:**
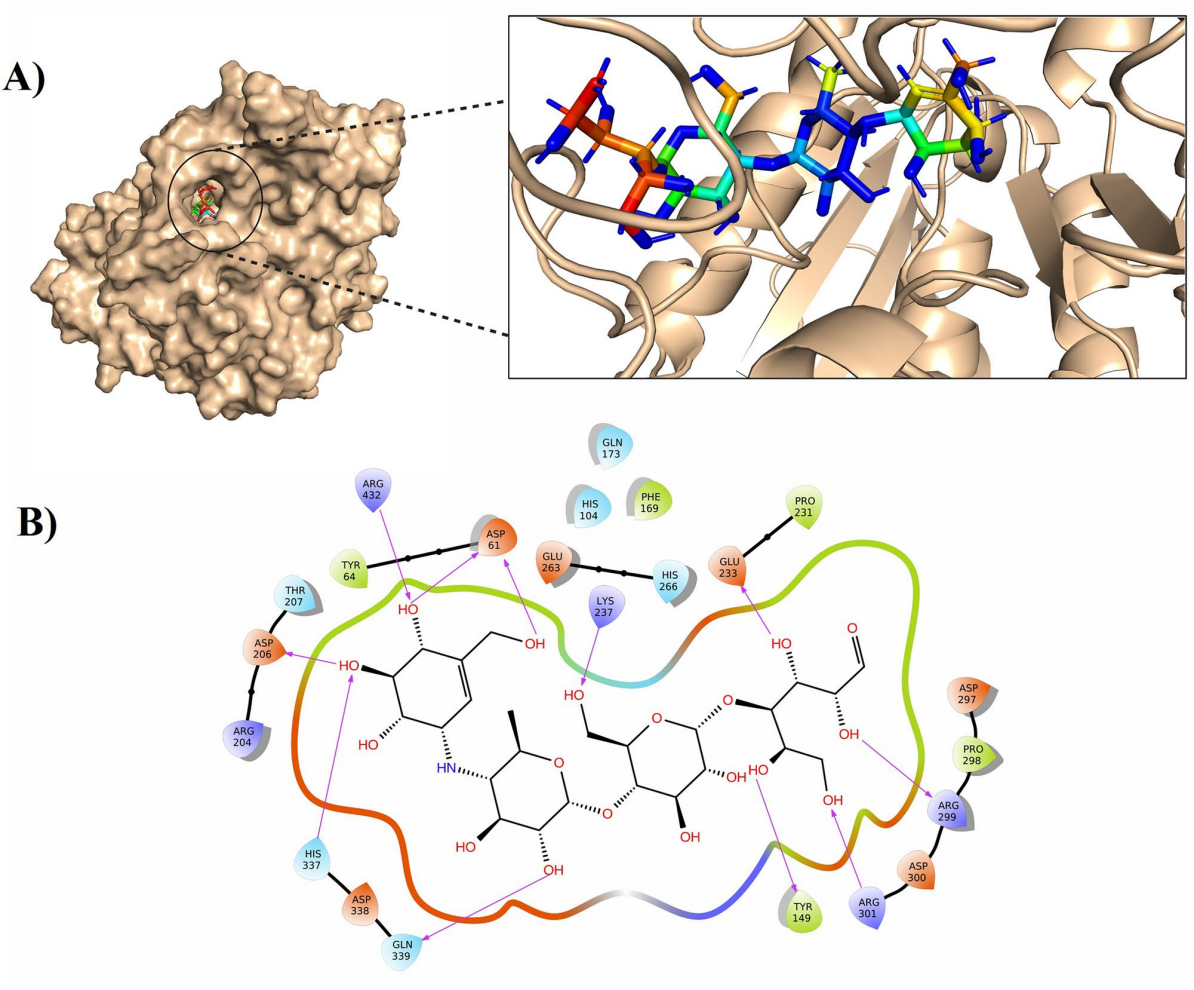
Illustrates the docked image of *C. albicans* α-glucosidase Acarbose complex interacting with active site residues (**A**) Three-dimensional docked pose; The protein structure is shown in wheat color surface representation (left): cartoon representation (right) and acarbose is shown as a rainbow stick representation. (**B**) Two-dimensional interaction. Hydrogen bond (purple arrow) with ASP206, Electrostatic Interaction (orange color) with GLU263 and ASP338.

Glycomimetic drugs, due to their strong affinity for α-glucosidase, offer a unique advantage in modulating carbohydrate entities within biological systems. This property enables precise control over broad-spectrum therapeutic effects, improved stability, enhanced bioavailability, extended half-life, targeted drug delivery, and enhanced formulation flexibility while minimizing potential toxicity^22^. All the 114 glycomimetic drugs approved by the FDA were retrieved from Drugbank. LigPrep by Schrodinger optimized and minimized all the ligands for docking. The prepared ligands were docked with α-glucosidase QSL-M1 with extra precision and further the glide score was analyzed (Table S2). From the glide scores generated, Acarbose came as one of the top-hit molecules with a score of − 11.47. Further, two-dimensional, and three-dimensional interaction analysis revealed that Acarbose interacts with the target by hydrogen (GLU263) and polar bonds (GLU263 and ASP338) with the conserved residues in the active site (Fig. 2). Acarbose is a known inhibitor of human α-glucosidase, and it is known for the breakdown of complex sugars into monomers. Because of this ability, it is targeted in type II diabetics to control blood glucose levels. Interestingly *C. albicans* α-glucosidase shows structural and functional similarity with human α-glucosidase with the same conserved catalytic amino acid residues in the binding pocket (ASP and GLU). Therefore, human α-glucosidase and acarbose interaction is taken as a positive control to compare the binding affinity of the *C. albicans* α-glucosidase acarbose complex. Hence, human α-glucosidase and acarbose are docked setting grid box around these conserved residues and the binding efficacy is scored as − 12.25 (Fig. S1). In both cases, it is evident that there is a strong hydrogen bonding between the protein and ligand.

α-glucosidase is crucial for disruption of fungal cell wall integrity characterized by defective cross-linking results in impaired adhesion and biofilm formation, reduced secretion of hydrolytic enzymes, modulation of immune recognition, and the impairment of filamentation^23^. Therefore, the study identifies Acarbose can be a potent inhibitor of α-glucosidase, highlighting its strong binding affinity.

#### Molecular simulation

**Figure 3:**
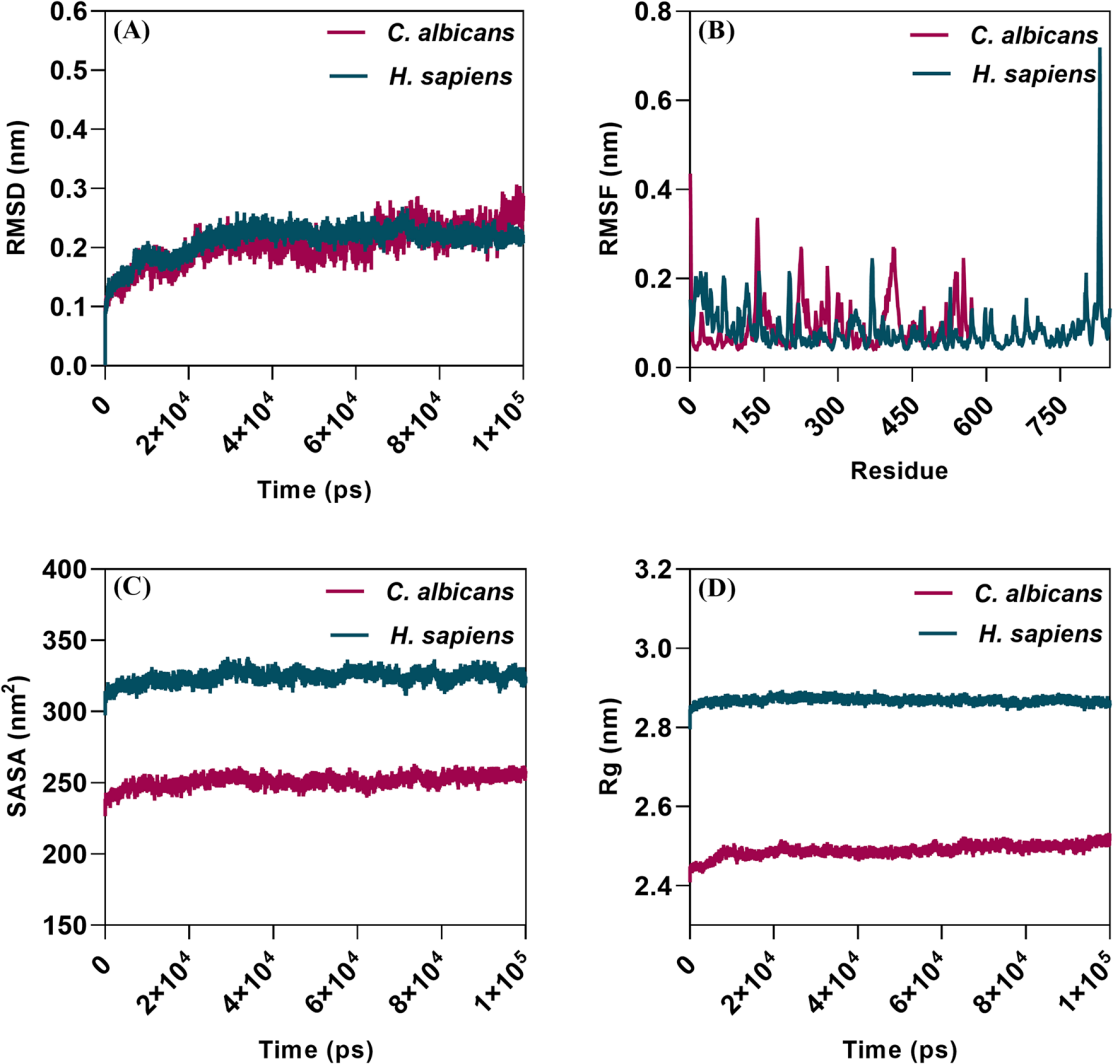
Molecular dynamics simulation of Human and *C. albicans* α-glucosidase-acarbose complex (**A**) RMSD; (**B**) RMSF; (**C**) SASA; (**D**) Rg. Pink color depicts the *C. albicans* α-glucosidase acarbose complex and the green colour depicts the Human α-glucosidase acarbose complex.

The MD simulations for the α-glucosidase-acarbose complex of *C. albicans* were analyzed to understand the complex’s energetics, dynamics, and stability^24^. Therefore, understanding the stability dynamics of the α-glucosidase-acarbose complex conducive to infection is determined by calculating the Root mean square deviation (RMSD), Root mean square fluctuation (RMSF), Radius of Gyration (Rg), and Solvent-accessible surface area (SASA). The RMSD calculations reveal that the α-glucosidase-acarbose complex of *C. albicans* was stable without any fluctuations as in humans where acarbose is a known inhibitor of α-glucosidase. In which, the equilibrium ranged between ~ 0.1–0.25 nm in Humans and ~ 0.1–0.3 nm in *C. albicans* (Fig. 3A**)**. Similarly, the RMSF calculations showed that the fluctuation range was very minimal in *C. albicans* occurring between ~ 0.05–0.42 nm. In the human α-glucosidase-acarbose complex, the fluctuations are between ~ 0.05–0.7 nm. This shows that the α-glucosidase-acarbose complex is stable (Fig. 3B). The Rg plot revealed that there is no fluctuation in the α-glucosidase-acarbose complex of *C. albicans* as in humans, and hence, the α-glucosidase-acarbose complex is structurally compact (Fig. 3C). The solvent-accessible surface area simulation also revealed the compactness of the α-glucosidase-acarbose complex. Again, no major fluctuations were noted in *C. albicans* and Human (Fig. 3D). In conclusion, molecular dynamics simulations underscored the robust stability and structural compactness of the α-glucosidase-acarbose complex in *C. albicans* Based on this foundation, it was subsequently subjected to in vitro analysis.

#### Effect of acarbose on planktonic and biofilm cells

**Figure 4:**
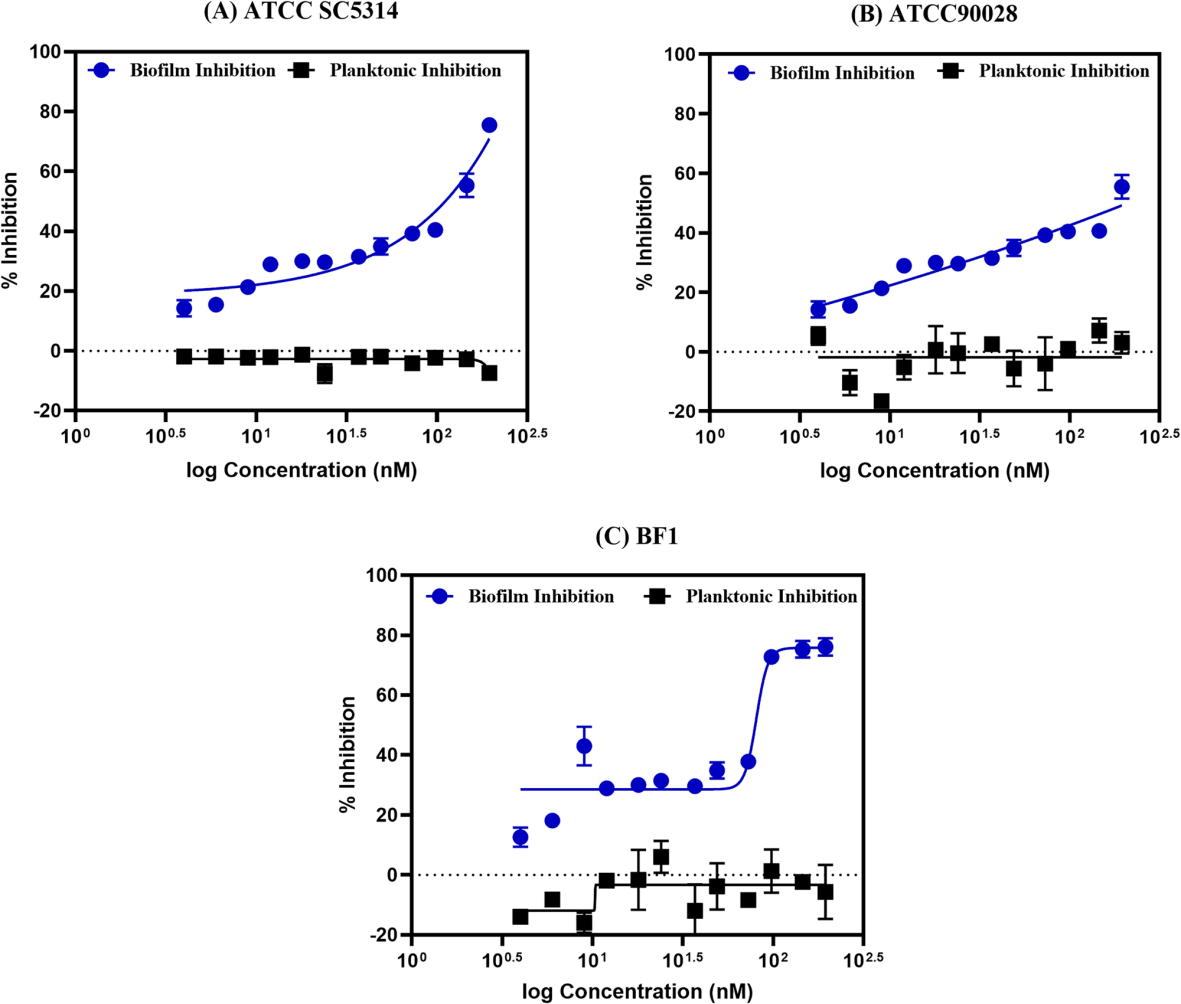
Illustrates the antifungal and antibiofilm activity of Acarbose against *C. albicans* at varying concentrations (**A**) SC5314; (**B**) ATCC90028; (**C**) BF1 (Clinical isolate). The dose–response curve was fit using a four-parametric logistic hill equation.

In Fig. 4, we illustrate the dose-response effects of Acarbose on the growth and biofilm formation of three distinct *C. albicans* strains within the tested concentration ranges. Of particular interest is the observation that Acarbose demonstrates no discernible effect on fungal growth within the specified experimental concentration range. A concentration-dependent biofilm inhibition was observed for all three strains considered, with an effective concentration range from 90 to 200 nM. Hence, we report for the first time, the off-target use of acarbose as an anti-biofilm compound against *C. albicans*. Previous reports on Acarbose showed biphasic behavior for *Mycobacterium smegmatis* where biofilm stimulation at lower concentrations (100 µg/mL) and biofilm inhibition at higher concentrations of 500 µg/mL were observed^25^. In our study, the reverse was observed where the biofilm inhibition was observed at an extremely low concentration of ~ 90 nM without affecting the fungal growth. Acarbose was explored for the antifungal activity against *Saccharomyces cerevisiae*^26^*,* our study extends the spectrum of Acarbose to be exploited for anti-virulence therapy against *C. albicans*. It should be also noted that the anti-diabetic effect of Acarbose is reported in the range of µM, typically more than 300 µM^27^. The anti-biofilm effect against *C. albicans* is far less in the range and thereby the anti-diabetic effect at the concentration of 90–200 nM is unlikely, limiting the crosstalk of acarbose between the human and *C. albicans* α-glucosidase.

#### Effect on filamentation and hyphal formation

**Figure 5:**
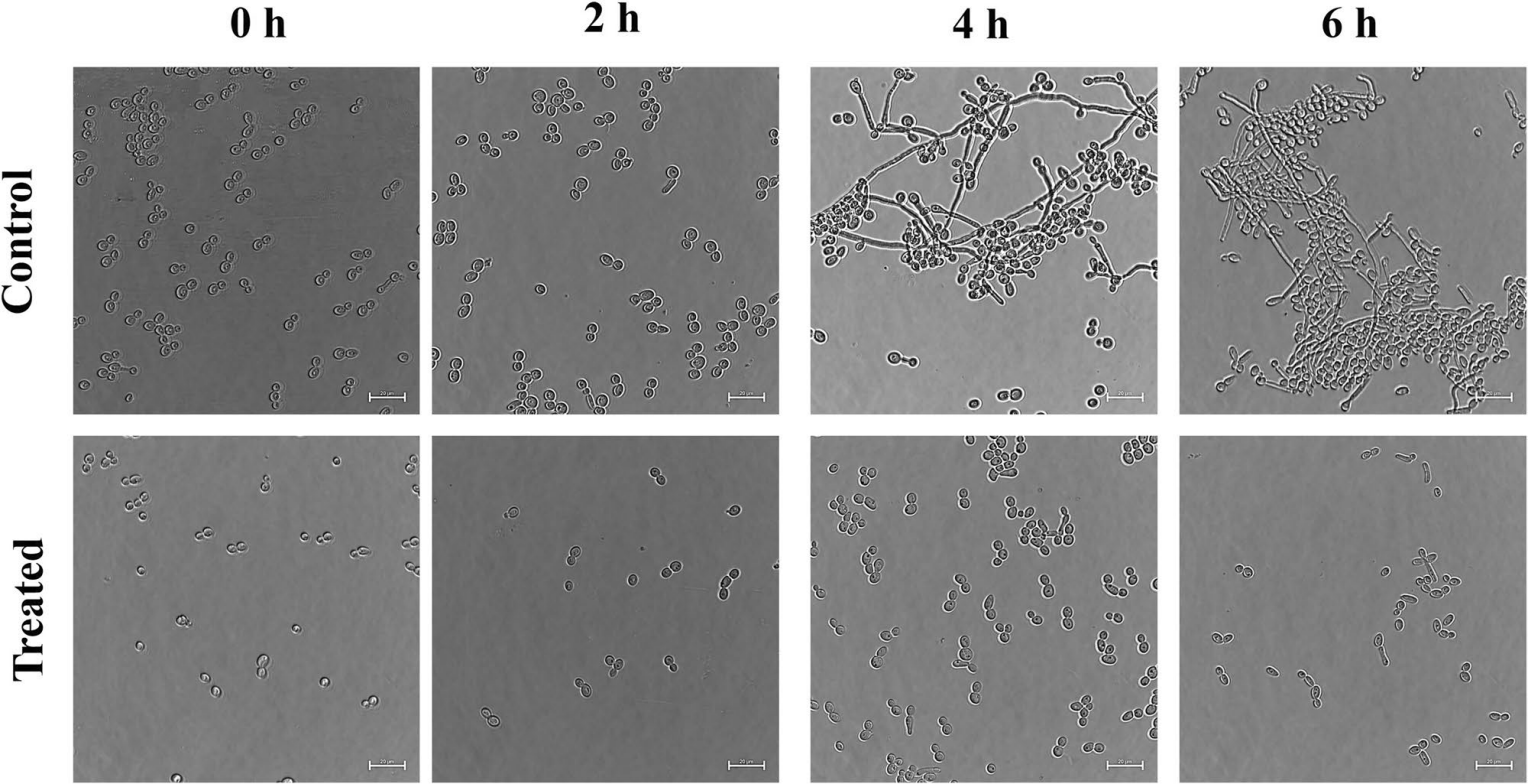
Illustrates the representative Germ tube inhibition kinetics of SC5314 at 200 nM of Acarbose. Phase contrast images were taken every 2 h till 6 h at 60× magnification to visualize the time-dependent inhibition of hyphal morphogenesis compared to the control. Until 6 h, the Acarbose treated cells were in the yeast form. The scale bar indicates 50 µm.

**Figure 6:**
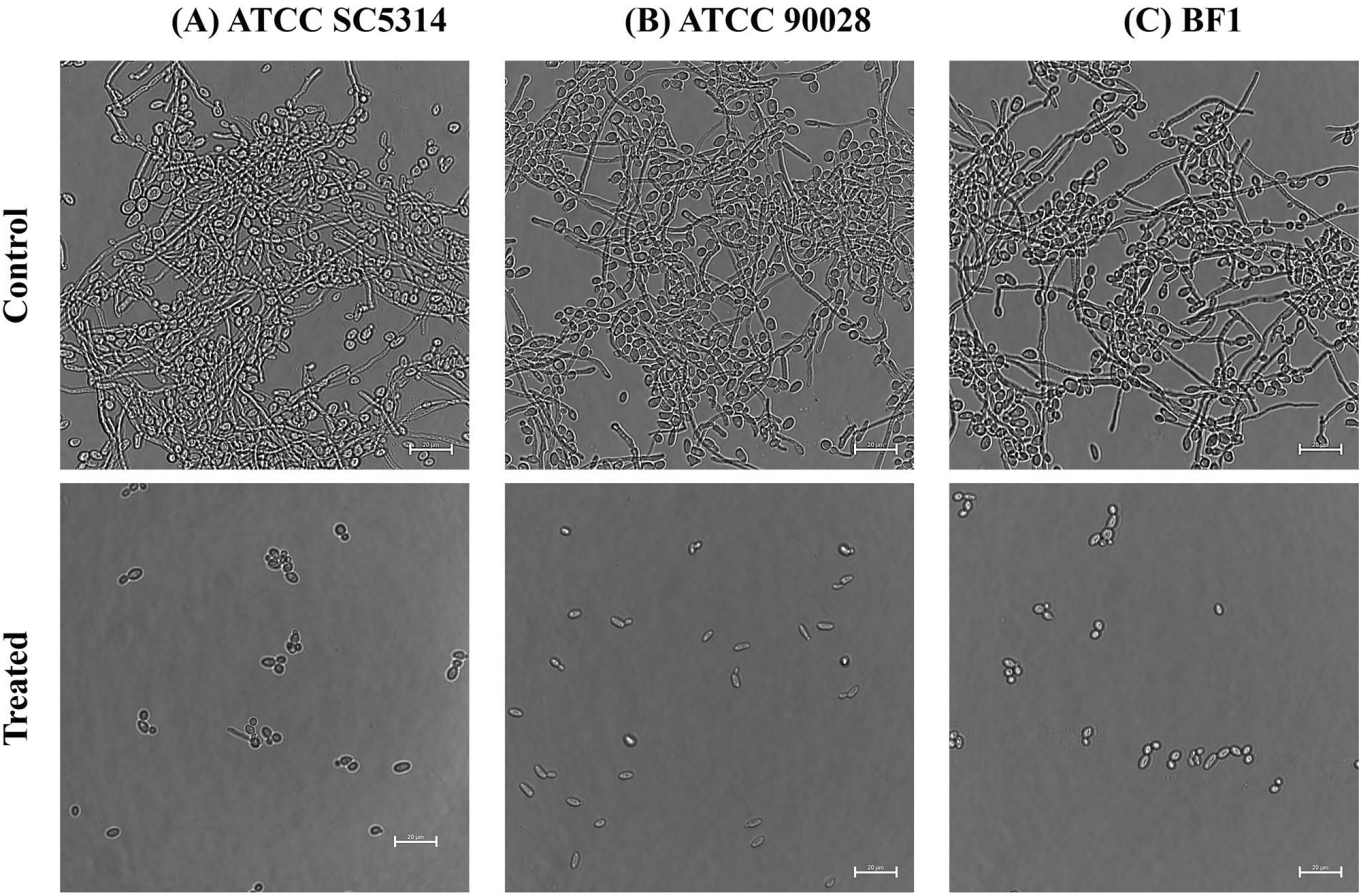
Illustrates representative Acarbose’s filamentation inhibition after 24 h. Phase contrast microscopic images were captured after 24 h at 60× magnification. The scale bar indicates 50 µm. (**A**) SC5314 (200 nM); (**B**) ATCC90028 (90 nM); (**C**) BF1 (90 nM).

The filamentous morphology, particularly observed in the hyphal forms of *C. albicans*, significantly amplifies its virulence and pathogenicity by enabling it to breach and invade host cells, thereby conferring increased resistance to immune responses^8^. A temporal assessment of germ tube formation was undertaken, encompassing intervals ranging from 0 to 6 h, with systematic observations recorded bihourly for three distinct strains: SC5314 (Fig. 5), ATCC90028 (Fig. S2A), and BF1 (Fig. S2B). Untreated cells show a transition from yeast cells to a well-established hyphal network in a time-dependent manner. In contrast, acarbose treatment from the beginning effectively hinders the morphogenic shift from yeast to hyphal forms. The hindering effect aligns well with the results obtained from the 24-h filamentation assay Fig. 6**.** These findings provide valuable insights into the potential of acarbose to disrupt the yeast-to-hyphal transition process, which is of great significance in *C. albicans* virulence and pathogenicity. The observed hindrance of yeast-to-hyphal transition in *C. albicans* through acarbose treatment aligns with the findings of the previous study on Mucin O-glycans as natural inhibitors of *C. albicans* pathogenicity. Both studies underscore the significance of disrupting the transition by targeting the filamentation^28^. The transition from yeast to hyphae was intricately linked to the adhesion and invasion processes. Subsequently, our evaluation aimed to elucidate the impact of acarbose on this transition.

#### Evaluating the effect of Acarbose on the adhesion and invasion of bladder epithelial cells

**Figure 7:**
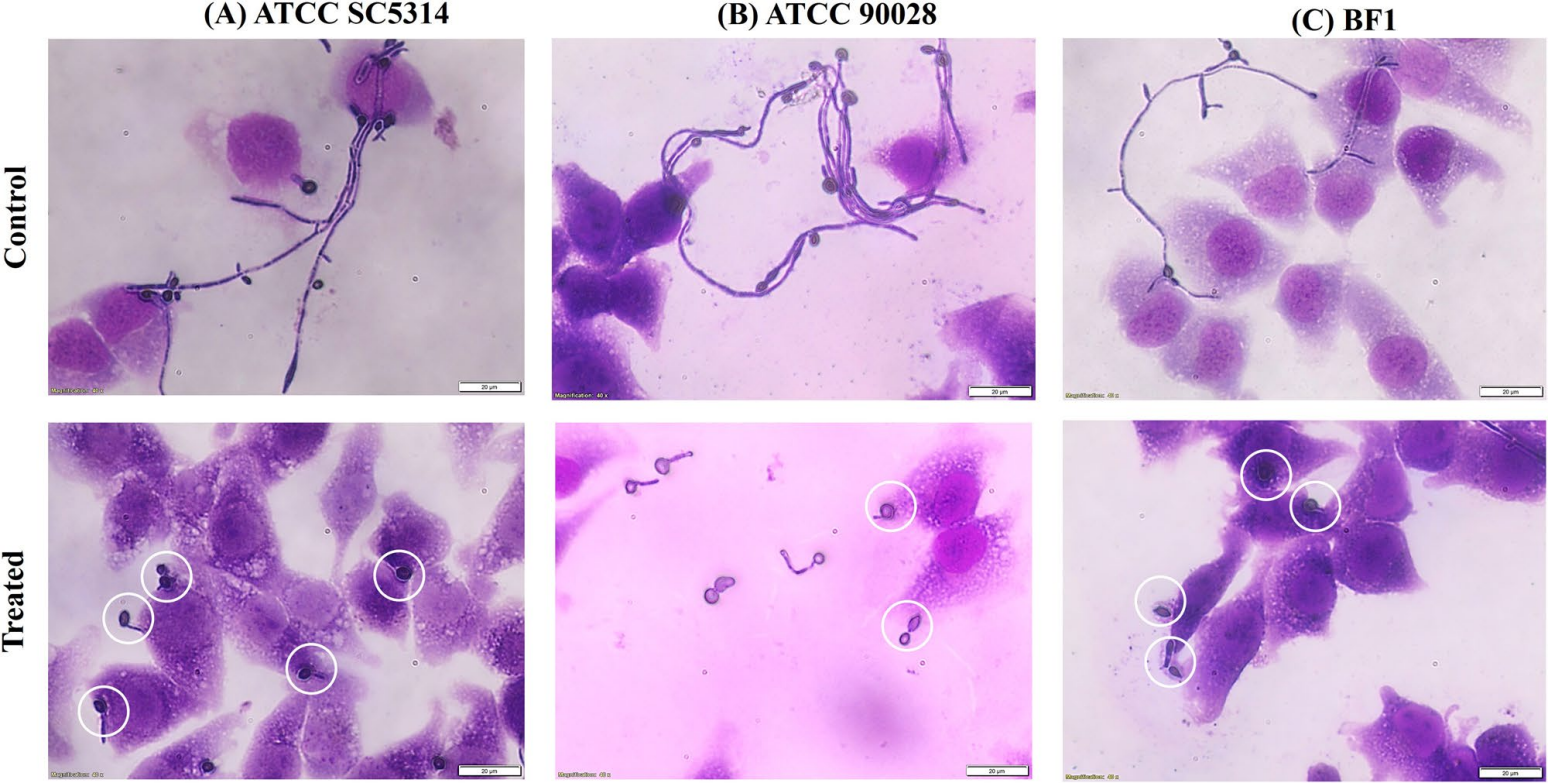
Illustrates representative Adhesion and Invasion Light microscopy images. Light microscopic images of the Giesma-stained infected monolayers of T24 bladder epithelial cell lines were taken at 40× magnification. The scale bar indicates 20 µm. Acarbose treatment inhibits the cell line model’s transition from yeast to hyphal morphogenesis.

*C. albicans* employ a detailed mechanism to adhere and further invade the host. The adhesion to the host epithelial cells marks the first step toward the infection process. Active adhesion is initiated in both yeast and hyphal forms, but hyphal forms which express more adhesins-related proteins are prone to stronger adhesion to the host^29^. Invasion of the fungal cells into the epithelial barrier also mandates the hyphal form^30^. Thus, to further prove the efficacy of Acarbose in reducing the adhesion and invasion of *C. albicans* T4 bladder epithelial cell line is used. Figure 7 shows the adhesion and invasion of *C. albicans* in the untreated conditions for the three strains. The highly filamentous form of *C. albicans* has adhered to and invaded the bladder epithelial cells. In contrast, the acarbose treatment led to shorter germ tubes leading to reduced adhered and invaded fungal cells. It should be noted that the concentration of Acarbose was maintained at 200 nM for the SC5314 strain and 90 nM for ATCC90028 and clinical isolate BF1. A visual examination also confirms the non-toxic effect of Acarbose against epithelial cells at the effective concentration range, as the mono-layer cells remain intact. The results above demonstrate that targeting *C. albicans* metabolism, specifically α-glucosidase, is an effective strategy. Acarbose, as an inhibitor of morphogenesis, adhesion, and invasion in *C. albicans*, may exert its effects by inhibiting various relevant genes. Identifying these genes is crucial for understanding the drug’s mechanism of action.

#### Acarbose downregulates the genes responsible for virulence and morphogenesis

**Figure 8:**
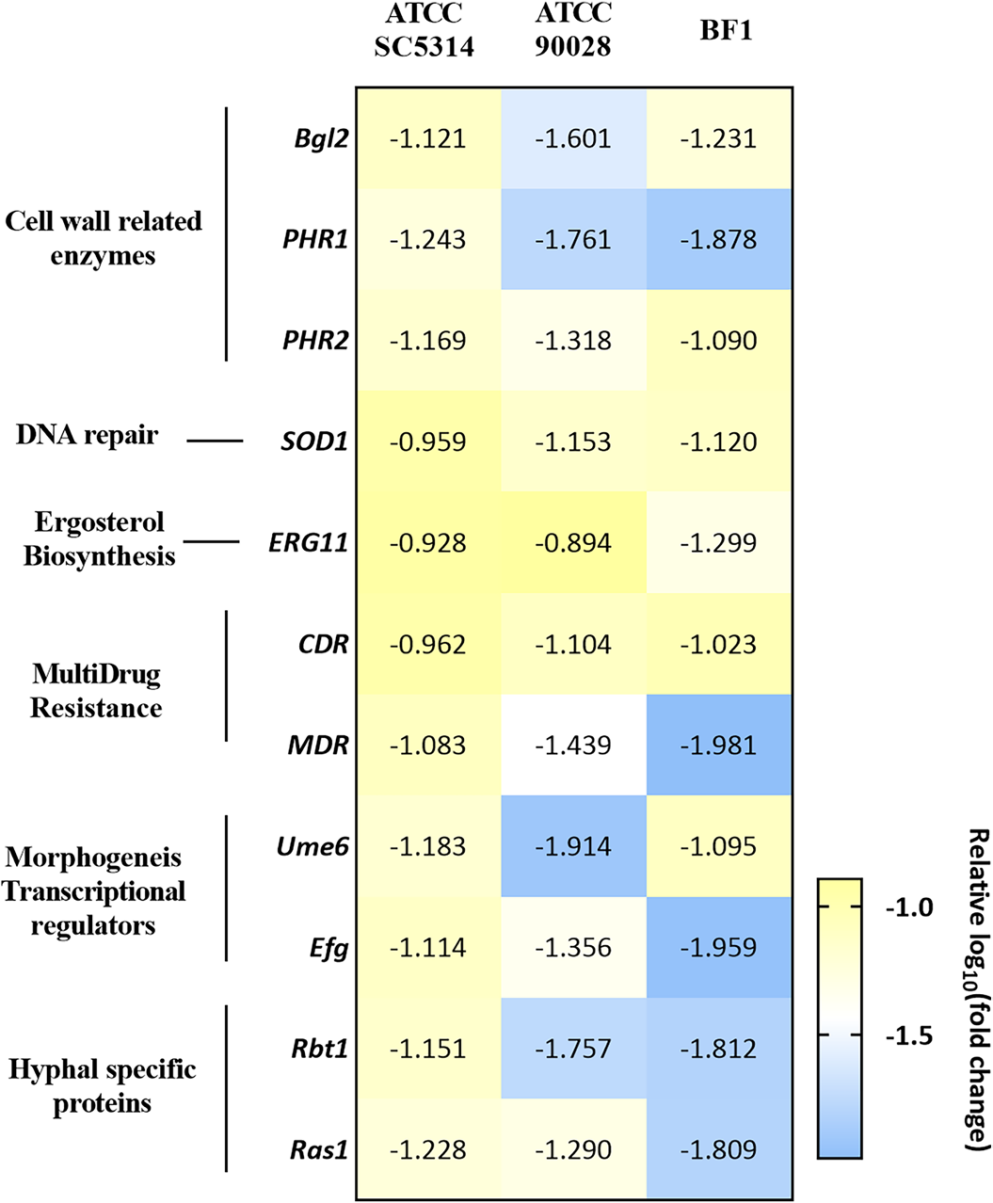
Illustrates qRTPCR analysis of biofilm, hyphal, and virulence genes of *C. albicans* treated with Acarbose using ITS as the housekeeping gene. Acarbose downregulated the genes responsible for the virulence in all three strains. The data was obtained in triplicates, and analysis was performed by the 2^−ΔΔCt^ method.

The effect of Acarbose on the biofilm, virulence, cell wall-associated and hyphal morphogenesis genes was evaluated using qPCR. Figure 8 depicts the gene expression of the genes from the three strains. α-glucosidase is a carbohydrate-splitting enzyme that is required for cell fitness and cell wall assembly^31^. Hence, the enzymes needed for cell wall biogenesis and virulence were considered to understand the mechanistic role. Further, the absence of α-glucosidase was shown to have impaired yeast to hyphal morphogenesis, shortened germ tube, and reduced host-fungal interaction. Hence, the genes responsible for morphogenesis, biofilm, and resistance were also considered. Figure 8 shows a strain-specific response to the gene expression profile in the specific genes considered. Interestingly, the clinical isolate was more sensitive to the acarbose treatment, where the genes were downregulated more than the standard reference strains. Nevertheless, all the considered genes were downregulated ~1.2 log fold in all three strains. *BGL2, PHR1*, and *PHR2* are the genes responsible for the cell wall structure and association with other interspecies interactions^32,33^. As expected, the hyphal-associated transcriptional regulators and specific proteins were downregulated more than the other genes. The downregulation of the hyphal-specific genes corroborates with the phenotypic assays of filamentation and germ tube inhibition. *UME6* and *EFG* are the transcriptional regulators responsible for the morphogenic shift^34^, and the regulators are downregulated for all three strains.

Additionally, the genes, *RBT1*, and *RAS1*, which are hyphal-specific proteins^34^, are downregulated at least 1.5log10 fold which clearly shows the diminished hyphal formation and hence the reduction of hyphal-specific proteins. Genes responsible for antimicrobial resistance – *CDR* and *MDR* were downregulated almost 2log_10_ fold. The other genes, *ERG1* and *SOD1*^34^, critical for virulence, are also downregulated with acarbose treatment. Thus, the acarbose treatment leads to impaired virulence and reduced yeast to hyphal morphogenesis.

#### Global transcriptomic effect of Acarbose on *C.albicans* using RNA sequencing analysis

**Figure 9:**
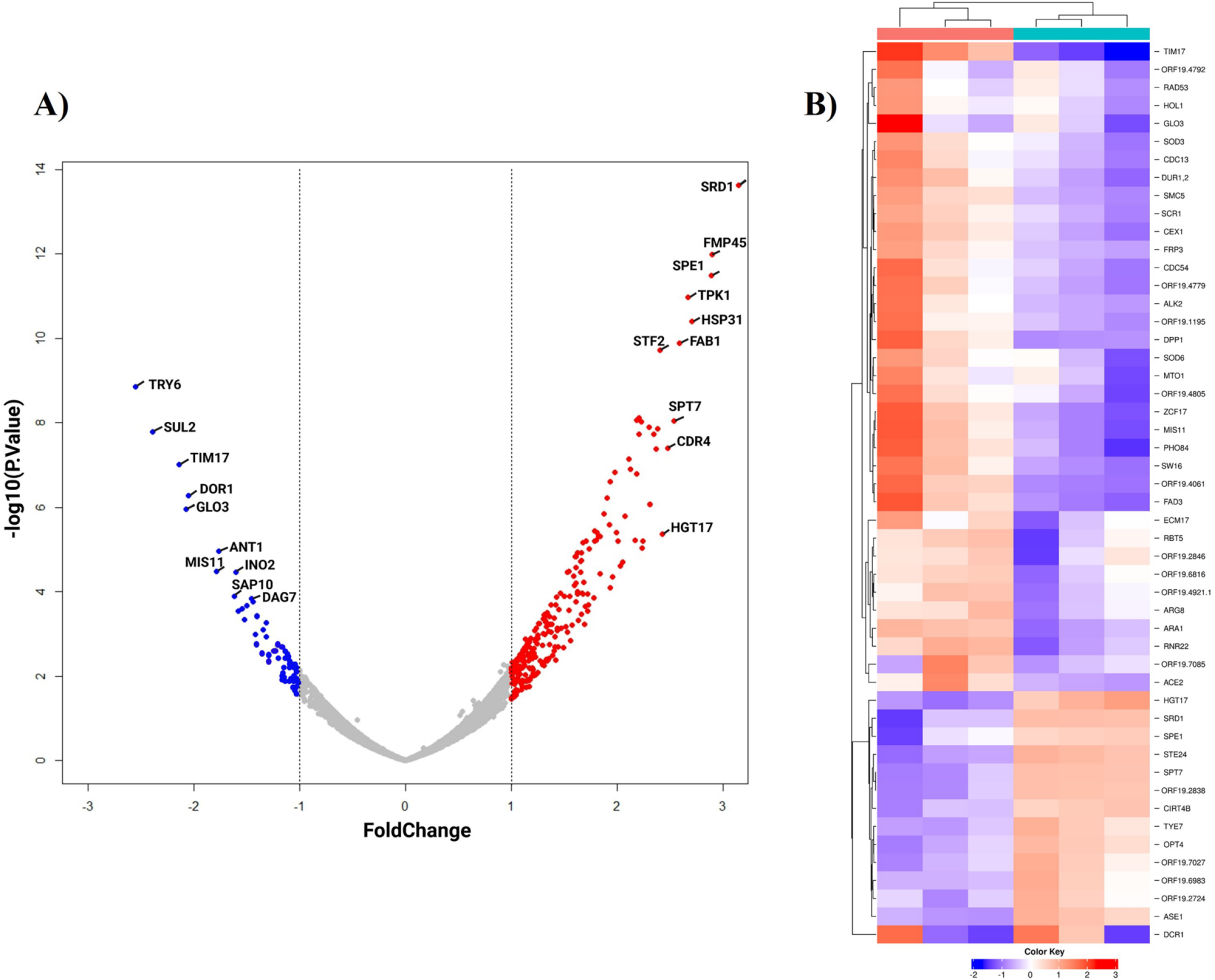
Transcriptomic analysis of *C. albicans* treated with acarbose. (**A**) Volcano plot of differentially expressed proteins. Red dots represent upregulated genes and blue color represents downregulated. (**B**) Heat map of top 50 differentially expressed genes.

To further understand the global transcriptomic changes, RNA Seq studies were carried out for the following conditions: 1. Untreated (control) *C. albicans* biofilm cells 2. 24 h Acarbose treated *C. albicans* biofilm cells. The Illumina sequencing produced the sequence reads from all samples, with more than 90% successfully mapped to the *C. albicans* strain SC5314 reference genome (GCF_000182965.3_ASM18296v3_genomic.gff). Principal-component analysis (PCA) and hierarchical clustering were applied to provide a visual representation of the transcriptomic similarities between samples treated with acarbose and the untreated controls. Samples from different conditions (presence or absence of Acarbose) clustered separately, while those from the same conditions clustered together, indicating a high level of correlation among samples, as well as distinctive transcriptome profiles. Analyses of the RNA sequencing data clearly indicate that acarbose has a profound effect on *C. albicans* gene expression leading to alterations in the transcriptome. For this analysis, genes that showed greater than a onefold (up or down) change in their level of expression were considered differentially expressed, and the cutoff for statistical significance used a Benjamini–Hochberg adjusted P value of < 0.05. A total of 322 genes showed a significant difference in expression between samples treated and those left untreated. Among 322 differentially expressed genes (DEGs), 247 were upregulated and 75 were downregulated in the compound-treated samples as shown in the volcano plot (Fig. 9A).

The heatmap presented in this study illustrates the expression patterns of the top 50 differentially expressed genes within treated and untreated clusters, revealing a pronounced divergence in gene expression profiles (Fig. 9B). Notably, the gene *SOD3*, responsible for encoding a cytosolic manganese-containing superoxide dismutase associated with oxidative stress protection, is significantly downregulated in the treated when compared to the untreated. Similarly, the gene *SOD6*, which encodes a copper-containing superoxide dismutase, also exhibits substantial downregulation in the treated cluster. The hierarchical clustering further underscores the close correlation between these two genes, implying shared functional relatedness. Likewise, the expression counts of other virulence-causing genes such as *PHO84, RBT5, CDC54, PHR1*, and other genes associated with *C. albicans* pathogenesis exhibit a significant downregulation, consistent with the previous findings, when compared to the untreated condition (add discussion).

**Figure 10:**
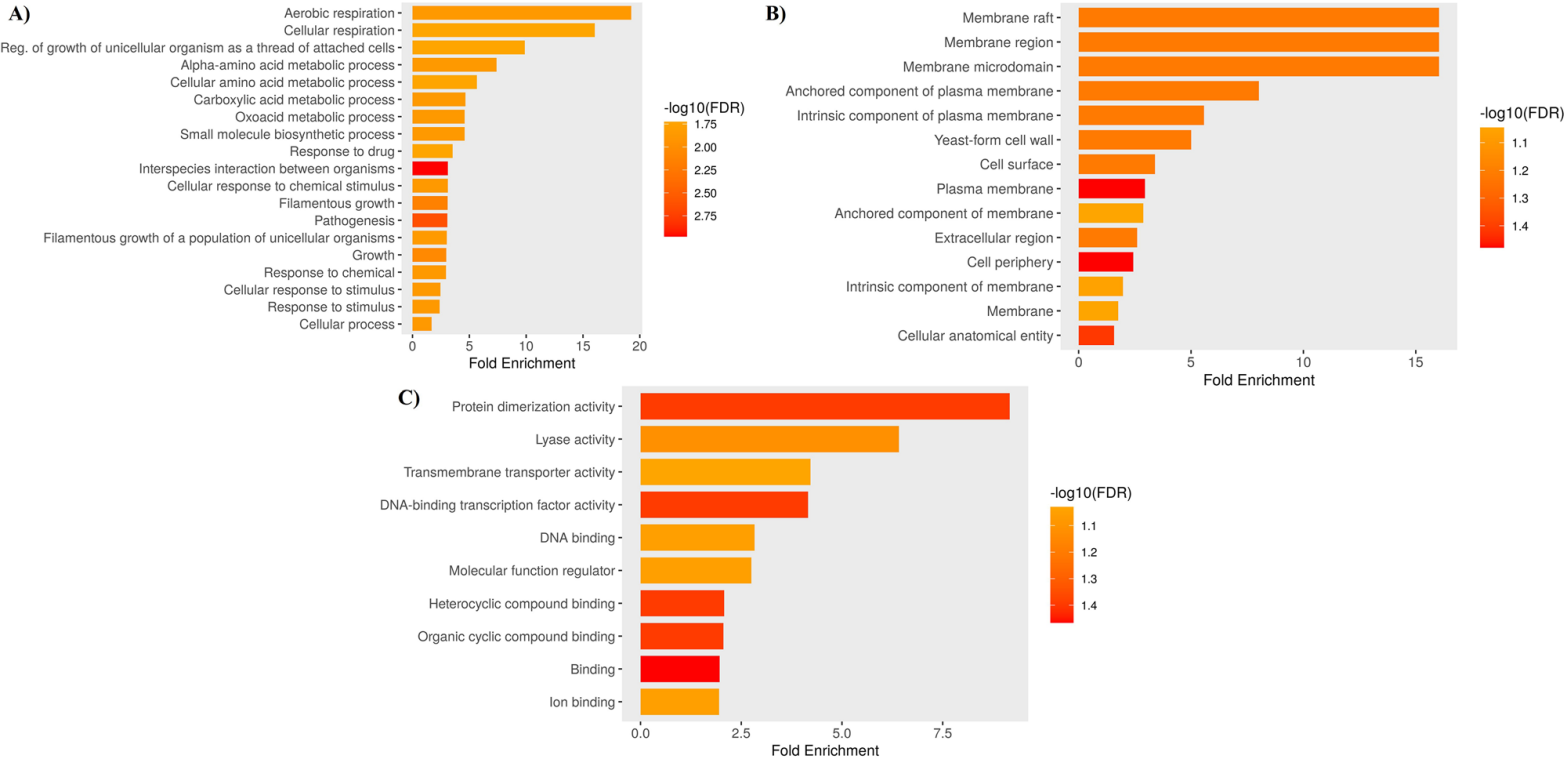
Gene ontologies enrichment analysis of the differentially expressed genes. Gene Ontology Term Finder tool ShinyGO was used for functional enrichment analysis, using default parameters. Valid results were considered with a *p*-value < 0.05. (**A**) Biological Process (**B**) Cellular Component (**C**) Molecular Function.

Gene Ontology (GO) analysis demonstrated the differentially expressed genes enriched in the GO term of cellular response to acarbose. The top 20 enriched pathways (with a *q*-value < 0.05) were illustrated for each Gene Ontology (GO) term, including Biological Processes (BP), Cellular Component (CC), and Molecular Function (MF), based on the differentially expressed genes (Fig. 10).The enrichment and upregulation of fundamental metabolic pathways, such as aerobic respiration, growth, carboxylic acid metabolism, and cell wall component synthesis specific to the yeast form, suggest that basic metabolism is minimally impacted, reducing the survival pressure on the organism . Similarly, membrane transmembrane transporter activity, cell surface, and plasma membrane component pathways are downregulated. These pathways play pivotal roles in nutrient acquisition, adhesion, immune evasion, and antifungal resistance, collectively contributing to the pathogenesis of *Candida* infections.

**Figure 11:**
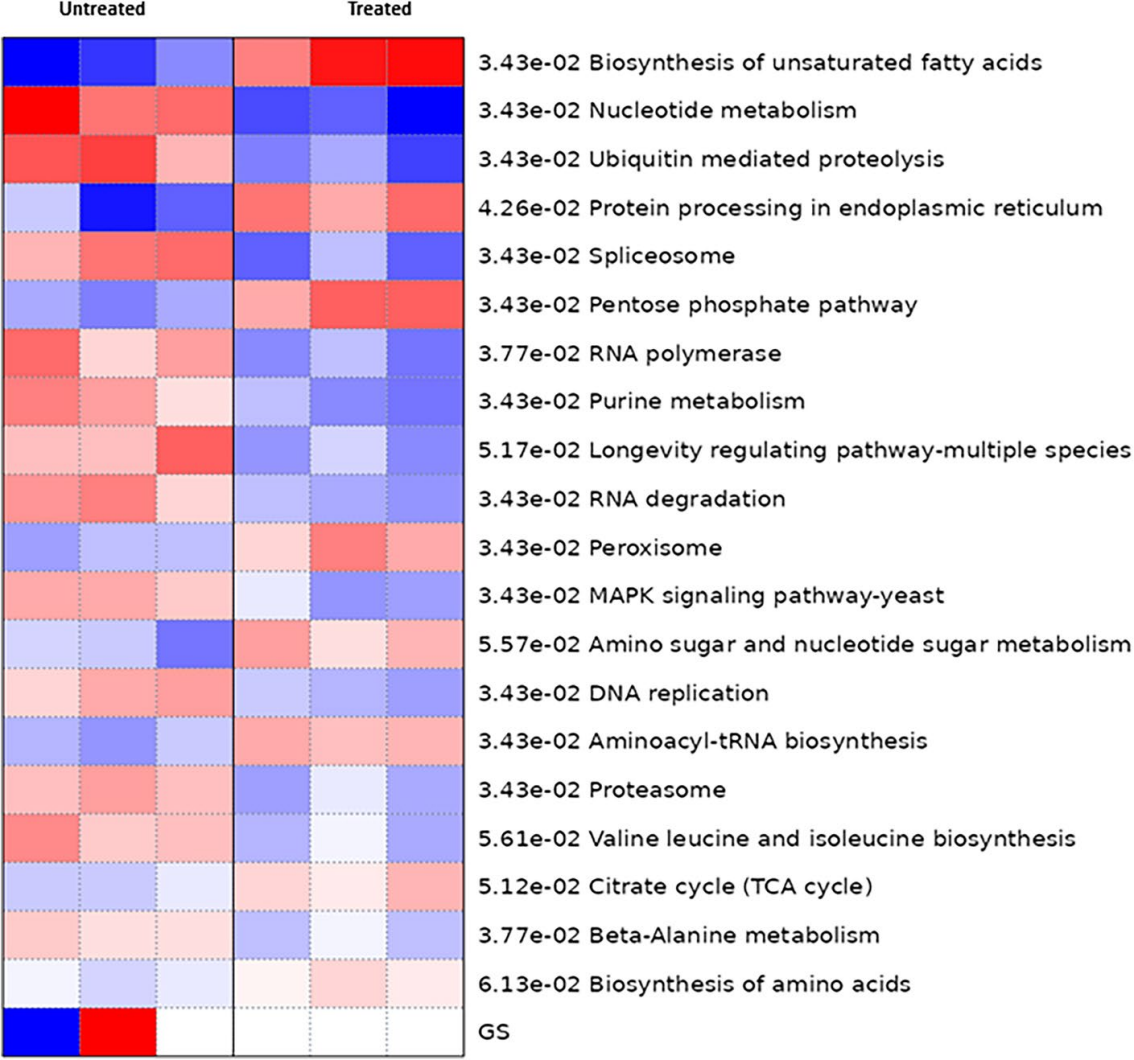
Pathway enrichment analysis of differential metabolites. (**A**) Kyoto Encyclopedia of Genes and Genomes (KEGG) pathway enrichment results of the differentially expressed genes. Red and blue indicate relatively activated and suppressed pathways, respectively. GS just indicates the color scale.

**Figure 12:**
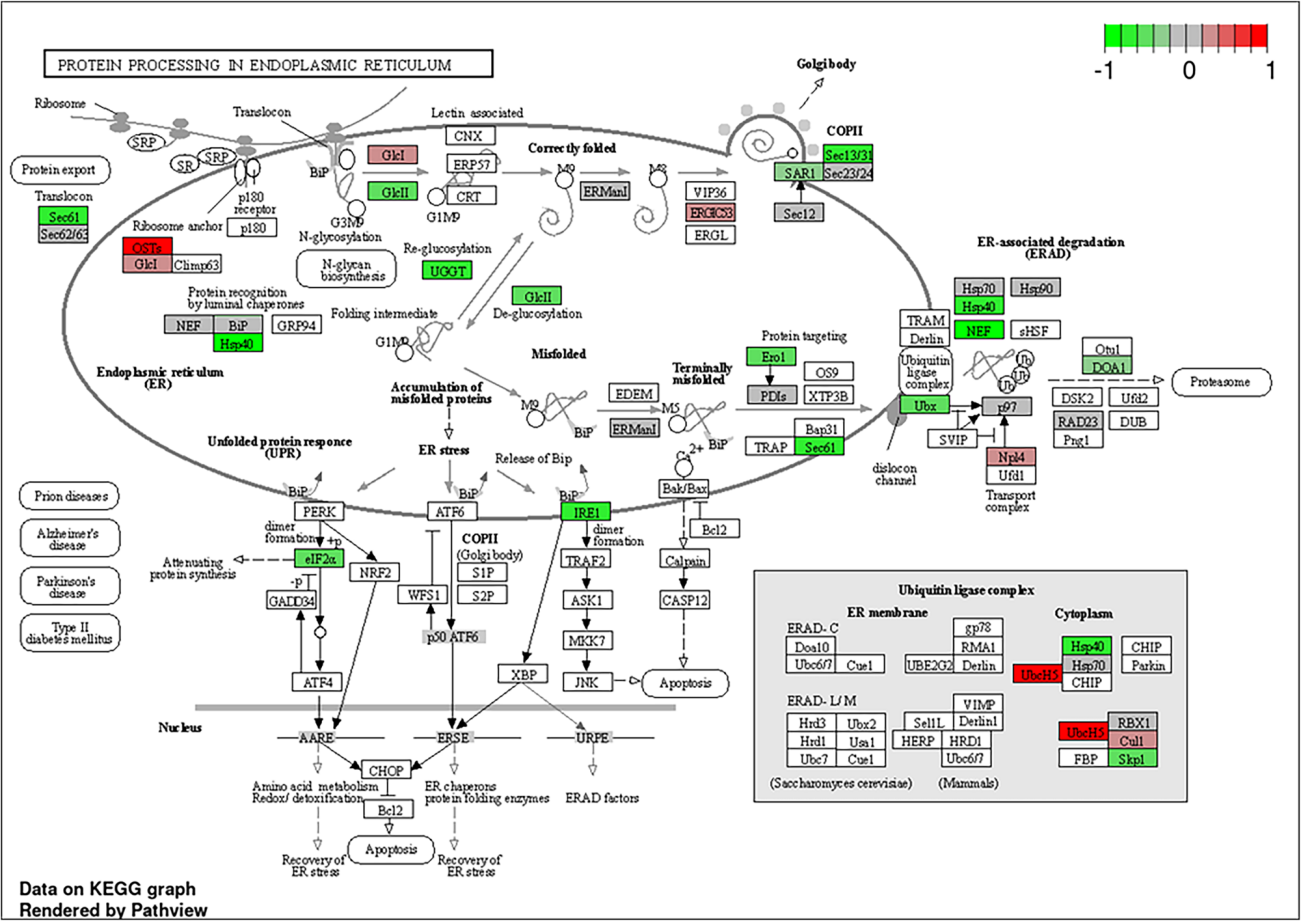
Pathway of Protein Processing in Endoplasmic Reticulum Enriched in pathway enrichment analysis. Image downloaded from iDEP1.1.

Further, the differentially expressed genes were primarily clustered in 20 KEGG pathways (Fig. 11). One of the notable KEGG pathways identified pertains to protein processing in the endoplasmic reticulum (Fig. 12). The endoplasmic reticulum (ER) is a cellular organelle responsible for the folding of proteins, aided by chaperones within its lumen. Newly synthesized peptides enter the ER through the sec61 pore and undergo glycosylation^35^. Properly folded proteins are then enclosed in transport vesicles, transporting them to the Golgi complex. Misfolded proteins remain in the ER lumen, forming complexes with molecular chaperones. Terminally misfolded proteins, identified by binding to BiP, are targeted for degradation through the proteasome in a process known as ER-associated degradation (ERAD)^36^. The accumulation of misfolded proteins in the ER induces ER stress, activating the unfolded protein response (UPR). In severe cases, the UPR’s protective mechanisms may prove insufficient to restore normal ER function, leading to cell death through apoptosis. Concurrently, ER stress responses are activated in *C. albicans* cells to cope with the demand for proper protein folding and secretion. This inter-relationship is highlighted by studies demonstrating that ER stress, triggered by factors such as cell wall stress, activates the unfolded protein response (UPR) pathway, which, in turn, influences the expression of genes involved in both ER and cell wall stress responses^37^.

The ER stress due to misfolding of proteins had cascading effects on numerous other KEGG pathways, including spliceosomes, ubiquitin-mediated proteolysis, biosynthesis of amino acids, purine metabolism, DNA replication, RNA degradation, protein export, and the MAPK signaling pathway in yeast. RNA splicing is a fundamental biological process governing gene expression in eukaryotic cells, and protein synthesis critically relies on the activity of spliceosomes. Proper maturation of gene transcripts through RNA splicing is essential to generate mature mRNA containing accurate protein-coding information. Consequently, RNA splicing holds a pivotal role in regulating gene expression^38^. The perturbation of this pathway underscores the significant impact of acarbose treatment on *C. albicans* gene expression. Another pivotal pathway is Ubiquitin-Mediated Proteolysis, which governs the regulation of various factors central to *C. albicans*’ pathogenicity. By controlling the degradation of intracellular proteins, this pathway allows *C. albicans* to adapt to the host environment and manipulate host cell processes to its advantage^39^. The significance of the MAPK Signalling Pathway lies in its role in enabling *C. albicans* to perceive and respond to environmental cues. This pathway is instrumental in the yeast’s ability to switch between yeast and hyphal forms, a crucial adaptation for tissue invasion and virulence, emphasizing its pivotal role in the pathogenic processes^40^.

**Figure 13:**
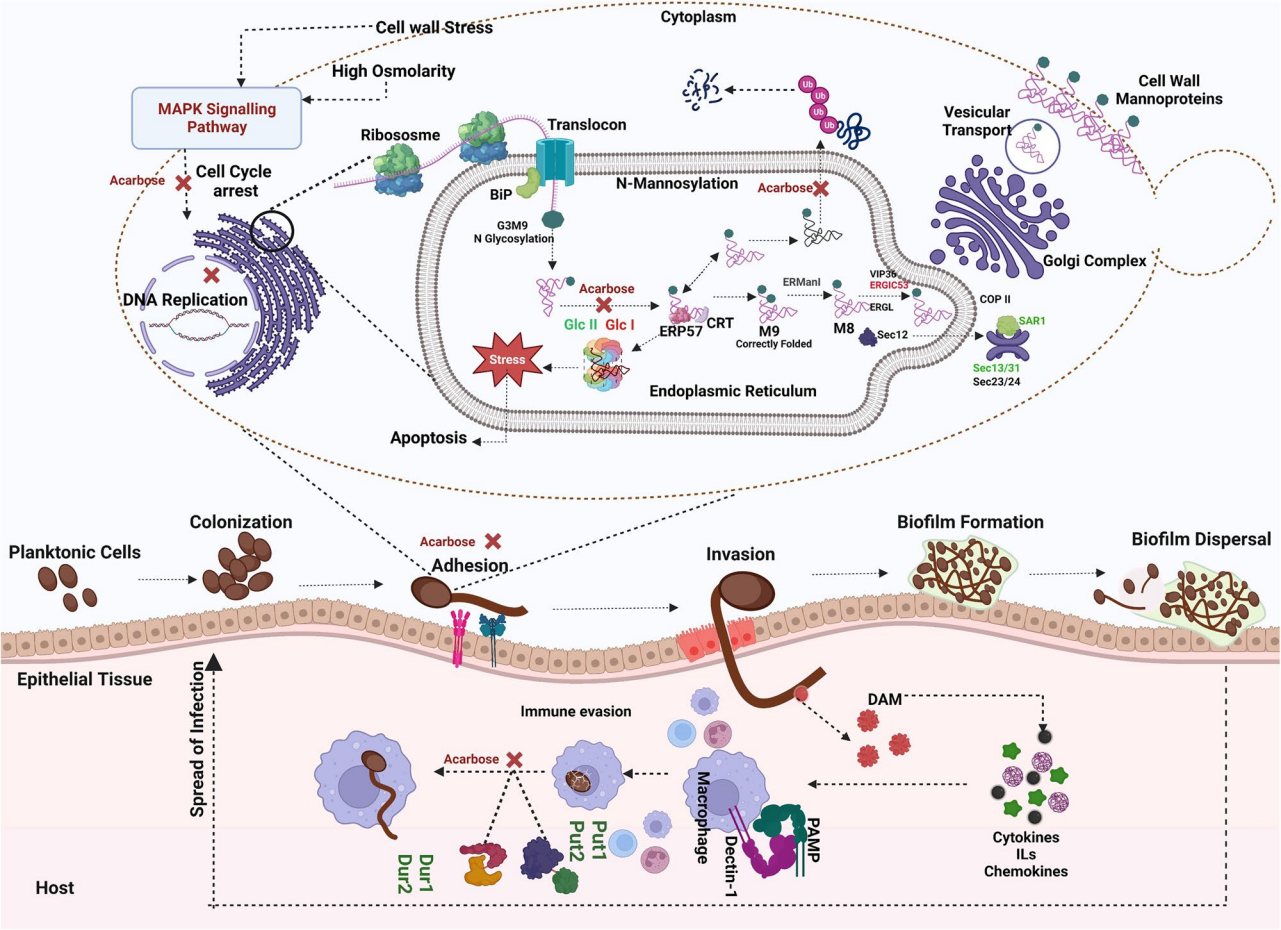
Mechanism of action of Acarbose against *C.albicans.* Dysregulation of protein processing induces endoplasmic reticulum (ER) stress, resulting in protein misfolding. Subsequently, this initiates cell wall stress, ultimately impacting cell wall architecture. Genes represented in green colour is downregulated and Red colour is upregulated. Created using biorender (https://www.biorender.com/).

In our transcriptomic analysis, we have observed a downregulation in the expression of genes associated with the processing of mannoproteins and the synthesis of cell wall-related proteins. This downregulation may result in the accumulation of unprocessed proteins within the endoplasmic reticulum (ER), inducing ER stress, heightened osmolarity, and cell wall stress. Subsequently, these conditions contribute to the downregulation of the MAPKK signalling pathway, initiating a cascade of events that impact the morphological change (Fig. 13). The coordination between protein processing in the ER and the synthesis of cell wall proteins underscores the importance of these dynamic cellular processes in maintaining *C. albicans* cell wall architecture. This, in turn, interferes *C. albicans*’ capacity to adhere to host tissues and evade immune responses, both critical steps in its pathogenesis^41^. This correlates with the previous results and hence proves our hypothesis.

## Materials and methods

### Homology modelling

The amino acid sequence of α-glucosidase from *C. albicans* was obtained from UniProt (ID: Q02751). Given the sequence homology between *C. albicans* α-glucosidase and Human α-glucosidase, the latter was used as a control. The 3D structure of Human α-glucosidase was retrieved from the Protein Data Bank. To predict the 3D structure of *C. albicans* α-glucosidase, the primary amino acid sequence was submitted to the AlphaFold2 server, which utilizes a neural network to directly predict the coordinates of all heavy atoms in the protein (Jumper et al., 2021). The models were generated and subsequently refined using the GalaxyRefine tool (www.galaxy.seoklab.org). The final protein structure was subjected to validation using the SAVESv6.0 (https://saves.mbi.ucla.edu/). Conserved residues, known for their catalytic activity, were identified based on scientific literature for both Human and *C. albicans* α-glucosidase. For further analysis, PyMol was employed to visually examine the binding pocket, while CASTp3.0 (http://sts.bioe.uic.edu/castp/) utilized the alpha shape method to identify topographical features, calculate area and volume, and determine the imprint. A grid box was constructed based on the conserved residues, facilitating additional investigations into the protein’s functional characteristics.

### Preparation of ligand library: glycomimetic drugs

The glycomimetic drugs approved by the US Food and Drug Administration are used for screening against the targets. The 114 approved drug molecules were retrieved from the DrugBank (https://go.drugbank.com/). These compounds were streamlined to treat various diseases. These compounds were subjected to preparation using LigPrep, by Schrodinger (Schrödinger, LLC, New York, NY, 2020).

### Molecular docking

The modeled and validated protein was refined, minimized, and optimized using Protein Preparation Wizard by Schrodinger (Schrödinger, LLC, New York, NY, 2020). Energy minimization, optimization, and refinement of the protein were performed to eliminate water molecules, protein atoms, and any steric clashes between amino acid residues. A grid box was centered on the conserved residues of the target, and the docking was performed using the extra precision (XP) docking algorithm implemented in Glide (Schrödinger, LLC, New York, NY, 2020). Based on the docking score the top lead-hit compounds have been screened.

### Molecular simulation

The α-glucosidase acarbose complex underwent molecular dynamics (MD) simulation using the GROMACS v4.5.5 (Groningen Machine for Chemical Simulations) package and GROMOS96 43a1 force field. To replicate the biological environment, the Single Point Charge 216 (SPC216) water model was applied, maintaining the system within orthorhombic boundaries measuring 10 × 10 × 10 nm^3^. Following the methodology and parameters outlined by Amala et al.^42^, simulations for both the human and *C. albicans* α-glucosidase acarbose complex were conducted and simulated for 100 ns. Trajectories were recorded at 2 ps intervals in both simulations. Analysis of trajectory files included evaluating Root Mean Square Deviation (RMSD), residual fluctuations, protein compactness, and solvent-accessible surface area. The trajectory values were then visualized and presented in a 2D graph using the Graph Pad Prism 8^43^.

### In vitro* evaluation: organism, media, and culture conditions*

The study utilized specific *C. albicans* strains, including the reference strains SC5314 and ATCC 90,028, along with the clinical isolate BF1, generously provided by Dr. Prasanna Neelakandan from the University of Hong Kong^44,45^. These strains were cultured on Yeast Extract Peptone Dextrose agar (YEPDA) at 37 °C under aerobic conditions. In preparation for each experiment, a single colony from the YEPDA plates was selected, suspended in Yeast Peptone Dextrose broth (YPD), and subjected to overnight incubation at 37 °C under aerobic conditions. Following incubation, the cultures were centrifuged at 6000 rpm for 10 min and underwent two washes with phosphate buffer saline (PBS). A standardized cell suspension was subsequently prepared by adjusting the Optical Density (OD_520_) to a range of 0.327–0.351, yielding a cell concentration of 1.5 × 10^7^ CFU/mL. Acarbose, sourced from Sisco Research Laboratories Pvt. Ltd in India, was used, and working concentrations of the compounds were achieved by dissolving them in YPD broth. All experiments were carried out in triplicate on three independent occasions.

### Susceptibility of planktonic cells

The minimum inhibitory concentration (MIC) of the top hit, Acarbose, was determined against the *C. albicans* strains ATCC90028, SC5314, and BF1. The strain was grown in Yeast Extract Peptone Broth (YEPD) as mentioned above. A wide range of concentrations ranging from 100 µM to 0.47 pM were considered for evaluation. The acarbose was serially diluted in 100µL of YPD and 10µL of the cell suspension culture was added to each well of a sterile 96-well plate and incubated at 37 °C for 24 h. Untreated standard cell suspensions with fresh YPD broth were considered as the control. The plates were then incubated in aerobic conditions at 37 °C for 24 h. Later, the growth inhibition was assessed by measuring OD_595_ using an Elisa plate reader (BioRad i-Mark). The MIC of acarbose is defined as the lowest concentration that inhibits the growth by ≥ 90% (for MIC90) or ≥ 50% (for MIC50) when compared to the control (untreated) using the formula ((control-test)/(control-blank)) *100^46–48^.

### Biofilm inhibitory effect

A 96-well microtiter plate assay was performed to characterize the biofilm-inhibiting activity of Acarbose. The biofilm biomass was measured using the Crystal Violet (CV) assay^49^. The test compounds were serially diluted in 100µL of YPD and 10µL of the cell suspension culture was added to each well of a sterile 96-well plate and incubated at 37 °C for 24 h. After incubation, the planktonic cells were carefully removed, and the biofilm was washed twice with PBS and stained with 0.1% crystal violet for 15–20 min. Then, 33% acetic acid was added, and the absorbance was measured at OD_595_ using an Elisa plate reader (BioRad i-Mark). The extent of biofilm inhibition was quantified using the formula ((control-test)/(control-blank)) *100^50,51^.

### Effect on filamentation: yeast to hyphal switch

#### Filamentation assay

The effect of the acarbose on filamentation was determined by allowing *C. albicans* to form hyphae under hyphal-inducing conditions. *C. albicans* strains with and without acarbose were grown in the spider medium (2% nutrient broth, 2% mannitol, 0.4% Dipotassium Hydrogen Phosphate, pH adjusted to 7.2) supplemented with 10% sterile FBS. After 24 h of incubation at 37 °C, 100 µL of the aliquots were centrifuged at 12,000 rpm for 10 min. The pellet was resuspended in 10 µL PBS and carefully mounted in a sterile coverslip to capture the fluorescence image at 40× magnification using the Nikon Eclipse Ts2 microscope.

#### Germ tube inhibition kinetics

Similarly, the germ tube inhibition kinetic study was performed with and without acarbose in similar hyphal-inducing conditions. 100 µL samples were collected every 2 h starting from the 0th hour to the 6th hour. The cells were pelleted and resuspended in 10 µL PBS and carefully mounted in a coverslip and the fluorescence images were captured at 40× magnification using the Nikon Eclipse Ts2 microscope.

### Cell adherence & invasion

The ability of the Acarbose to inhibit the *C. albicans* adherence onto the bladder cell lines was evaluated. Bladder epithelial cells share similarities with other mucosal surfaces where *C. albicans* can cause infections, such as the gastrointestinal, genito-urinary tract and respiratory tracts^52–54^. Briefly, the T24 cell lines were grown in the 6-well tissue culture plates till they reached 80–90% confluency in DMEM containing 10% FBS having sterile coverslips. In parallel, *C. albicans* strains were grown till the exponential phase. The monolayer of T24 cells was infected with *C. albicans* log phase cells. Acarbose was added at a concentration of 90 nM for ATCC90028 and Clinical isolate BF1; and 200 nM for SC5314 and the cells were incubated for 1 h at 37 °C. After incubation, the media was removed and washed with sterile PBS thrice to remove the unadhered fungal cells. After the washing step, the cells were fixed with 50% ice-cold methanol and incubated for 30 min. The coverslips were air-dried and stained for 30 min with Giemsa stain. The cells were examined using a microscope (Olympus BX43) with 40× magnification. For each sample, at least 5 images were captured.

### Gene expression analysis using qRT-PCR

The changes in *C. albicans* gene expression after treatment were observed using qRT-PCR analysis. Biofilms were developed with and without acarbose on sterile 6-well plates and incubated under aerobic conditions at 37 °C for 24 h. Then, the planktonic cells were removed, and biofilms were washed twice with PBS, scraped, and collected. The samples were then centrifuged at 14,000 × g for 10 min and the supernatant was discarded. The pellet was used to extract the RNA using the Promega SV Total RNA isolation system (Promega Corporation, Madison, Wisconsin, USA) as per the manufacturer’s guidelines. The purity and concentration of the RNA were measured using Nanodrop (Thermo Scientific, USA). The RNA was then converted into cDNA using cDNA Reverse Transcription Kit (Applied Biosystems, Foster City, California, USA) using the manufacturer guidelines and stored at − 20 °C. The gene expression was analyzed by qRT-PCR using 18srRNA^55^ as the housekeeping gene and the relative gene expression (fold change) was determined by 2^−ΔΔCT^ method^56^. The primers used in this study have been tabulated (Table S3).

### Gene expression studies: RNA seq analysis

The total RNA from the treated and untreated cells was extracted using a HiMedia Bacterial RNA isolation Kit. For each biological replicate, the cells were pooled from 4 replicate samples of each condition and RNA was extracted. The concentration and the purity of the total RNA were quantified using NanoDrop™ 2000 (Thermo Scientific, USA) was done to evaluate the purity of isolated RNA and saved at − 80 °C for the further steps. Libraries were prepared as per the modified NEBNext RNA Ultra II directional protocol. Cytoplasmic and Mitochondrial Ribosomal RNA were removed using biotinylated, target-specific oligos combined with rRNA removal beads. After purification, under high temp, RNA was fragmented using divalent cations. Reverse transcriptase and random primers were used in the cDNA synthesis process. The conversion of cDNA to double-stranded cDNA with uracil. USER enzyme was used in the digestion of the second strand and preservation of strand specificity. The digested single strands are enriched by PCR and then AMPure bead purification for cDNA library preparation. The prepared cDNA libraries were sequenced using Illumina HiSeqX/Novaseq to generate 2 × 150 bp reads/sample.

UseGalaxy web server (https://usegalaxy.eu/) was used to perform the analytical procedure of differential expression analysis. Sequence read archives (SRA) files were uploaded and converted into FASTQ files. FASTQC was used to assess the quality of reads at each step. Trimmomatic was used to remove low-quality sequences and adapter sequences from the data. HISAT 2 was used to align reads with the reference genome of *C. albicans* SC5314 (GCF_000182965.3_ASM18296v3_genomic.gff). Reads per gene frequency were determined by using FeatureCounts. The Limma Voom tool was used to normalize and exhibit differential gene expression. Filtration of insignificantly expressed genes was carried out by limiting the adjusted p-value to 0.05 and absolute fold change, FC > 1. Differentially expressed genes were visualized by creating volcano plots (via R studio), and Heatmap (via iDEP). Finally, the gene ontology studies were carried out using ShinyGO (http://bioinformatics.sdstate.edu/go/) and KEGG pathway analysis through iDEP1.1 (http://149.165.154.220/idep11/).

### Statistical analysis

All the assays were performed in biological and technical replicates and were analyzed using GraphPad Prism 8.0.2 software. Unpaired student t-test was performed to compare the significance between the control and treated groups. qRT-PCR data were analyzed and the log2 values of the 2^−ΔΔCt^ values were used to plot the heat maps and bar graphs.

## Conclusions

In this study, the antifungal activity of pseudo-sugar acarbose with a novel mechanism of action targeting α-glucosidase in *C. albicans* causing severe candidiasis was accomplished. The interaction between the α-glucosidase-acarbose complex was proven stable by in silico studies with docking and simulation. Acarbose interacts with α-glucosidase in the highly conserved residues in the binding pocket and the interaction is stable even in the dynamic state. Interestingly, acarbose was also successful in inhibiting the formation of biofilm by *C. albicans* without exerting survival pressure on the organism. These findings pave the way to investigate the effect of acarbose on the virulence of *C. albicans* especially adherence and hyphal formation. Acarbose was successful in diminishing the hyphal formation in hyphal-inducing conditions and adherence to the bladder epithelial cells. The gene expression studies also corroborate with the previous interpretation by downregulating the major virulence-causing genes in the *C. albicans.* The global transcriptomic changes in *C. albicans* were also assessed using RNA sequencing and major pathways leading to the hyphal development were observed to be downregulated. Through our comprehensive studies, we show that Acarbose can be a potent lead molecule for antifungal development. Further, synergy studies with current antifungals and in vivo studies are under steady progress to prove the efficacy of Acarbose in reducing the antifungal concentrations and thus, resistance development.

### Supplementary Information

Supplementary Information.

## Data Availability

The datasets generated during and/or analysed during the current study are available from the corresponding author upon reasonable request.
