# Supplementary material for: Mitigating candidiasis with acarbose by targeting Candida albicans α-glucosidase: in-silico, in-vitro and transcriptomic approaches
Source: Sci Rep. 2024 May 24;14:11890. doi: 10.1038/s41598-024-62684-x (PMC11126738; doi:10.1038/s41598-024-62684-x)
Supplement: Supplementary file 1 — Supplementary Information. [file 41598_2024_62684_MOESM1_ESM.docx]

**Supplementary Information**

**Mitigating Candidiasis with Acarbose by targeting *Candida albicans* α-Glucosidase: *In-silico, In-vitro* and Transcriptomic Approaches**

Helma David^1^, Sahana Vasudevan^1,2*^, Adline Princy Solomon^1*^

*^1^Quorum Sensing Laboratory, Centre for Research in Infectious Diseases (CRID), School of Chemical and Biotechnology, SASTRA Deemed to be University, Thanjavur, 613401, India.*

*^2^Institute for Stem Cell Science and Regenerative Medicine (inStem), Bangalore 560065, India*

Email IDs: [adlineprinzy@sastra.ac.in](mailto:adlineprinzy@sastra.ac.in) , [savvysahana@gmail.com](mailto:savvysahana@gmail.com)

**Figure S1:** Illustrates the docked image of Human α-glucosidase Acarbose complex interacting with active site residues (A) Three-dimensional docked pose; The protein structure is shown in wheat color surface representation (left): cartoon representation (right) and acarbose is shown as a rainbow stick representation. (B) Two-dimensional interaction. Hydrogen bond (purple arrow) with ASP443 and ASP542; Electrostatic Interaction (orange color) with GLU446.


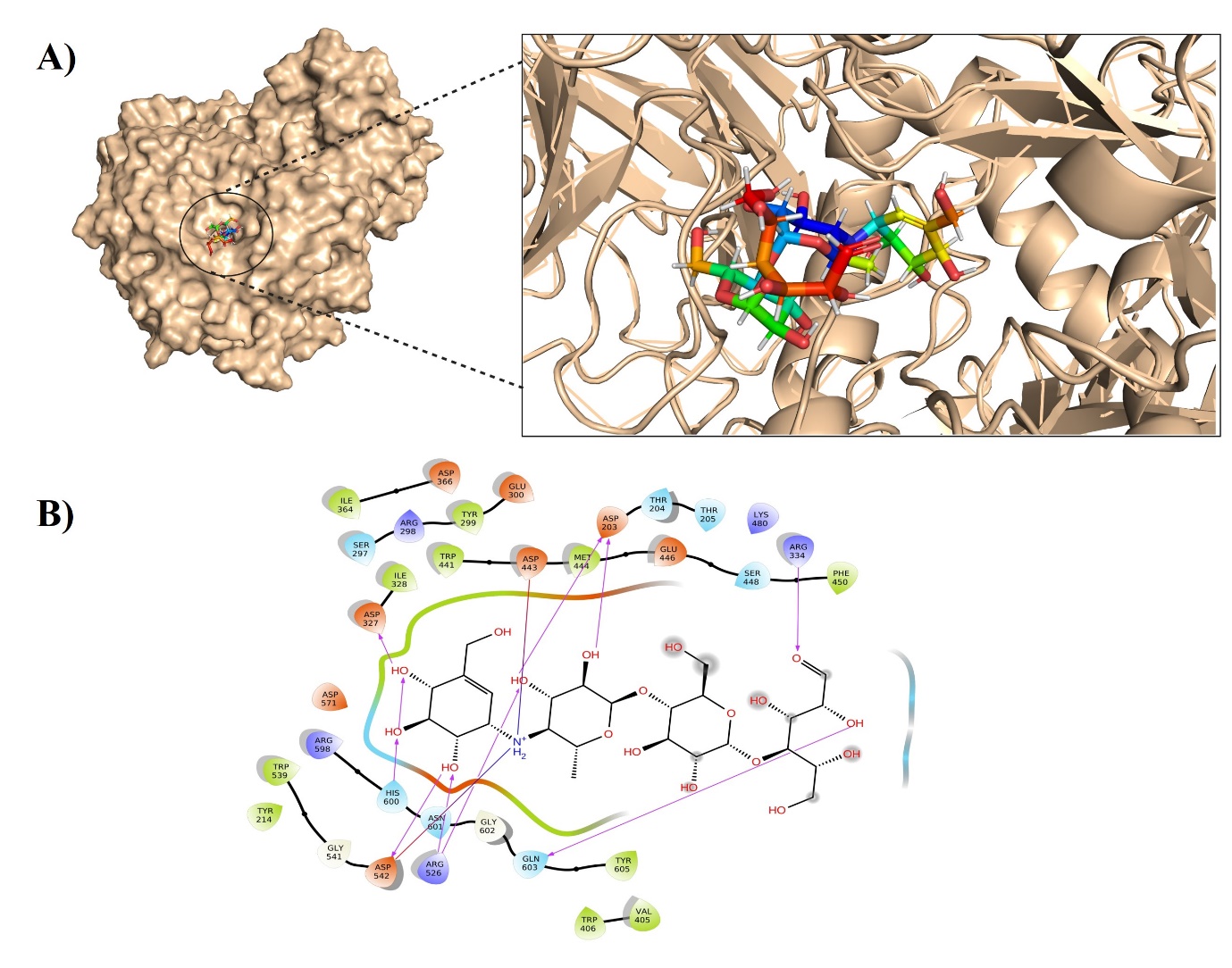


**Figure S2:** Illustrates the Germ tube inhibition kinetics of **A)** ATCC90028 and **B)** Clinical isolate BF1 90 nM of Acarbose. Phase contrast images were taken every 2 h till 6 h at 60 X magnification to visualize the time-dependent inhibition of hyphal morphogenesis compared to the control. Until 6 h, the Acarbose treated cells were in yeast form. The scale bar indicates 50 µm.


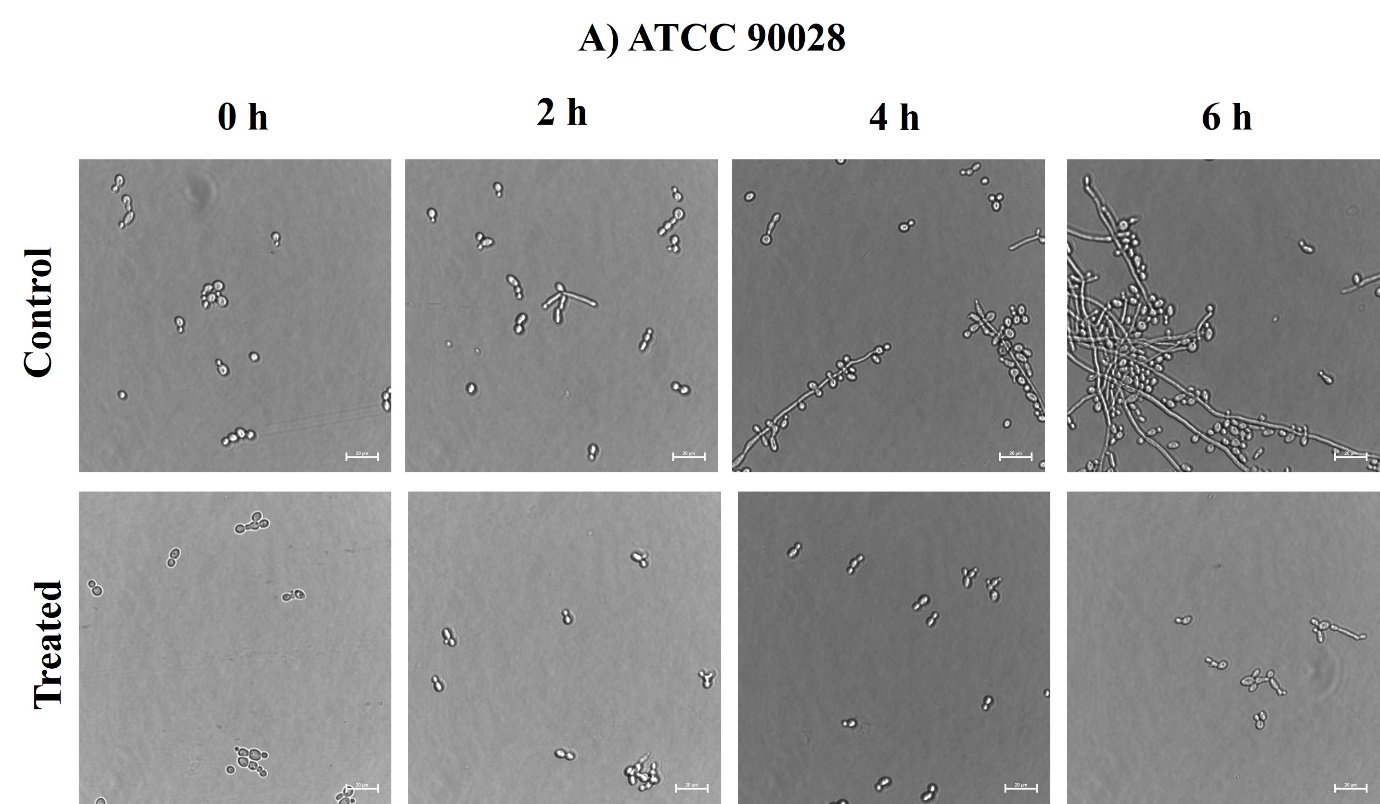

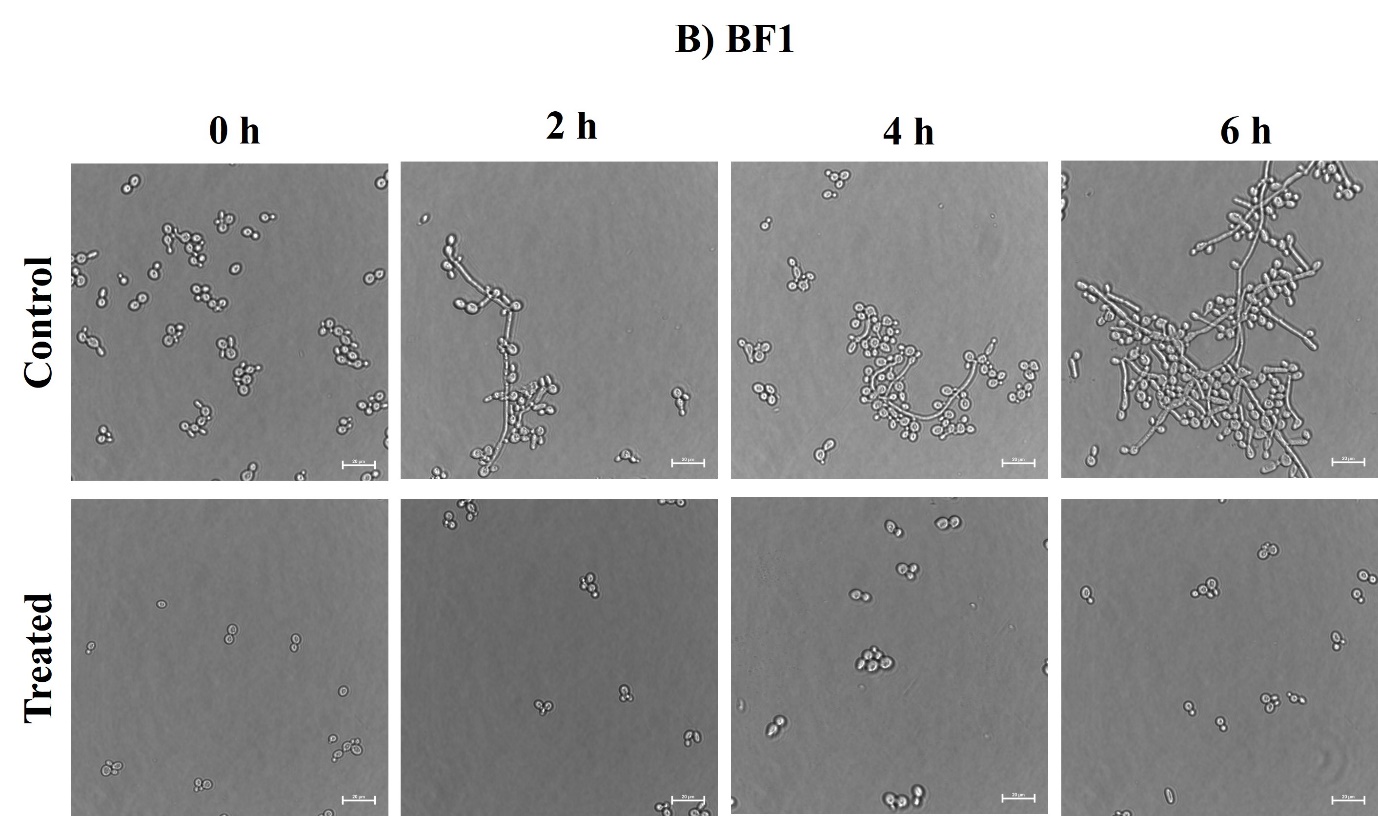


**Table S1:** AlphaFold2 generated Models of *Candidal* α-glucosidase.

| **Sl. No:** | **Model** | **Structure** | **Quality Factor** | **Ramachandran Plot** | |
| --- | --- | --- | --- | --- | --- |
|  |  |  |  | **Residues in the most favorable region (%)** | **Residues in Disallowed Region (%)** |
| 1 | QSL-M1 | 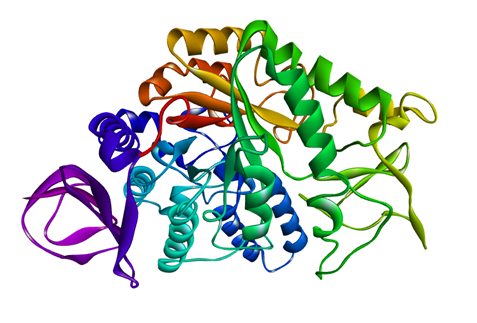 | 93.9 | 95.9 | 0.2 |
| 2 | QSL-M2 | 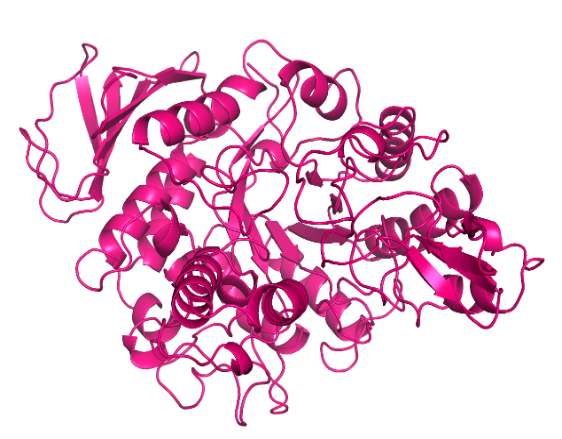 | 92.7 | 95.5 | 0.2 |
| 3 | QSL-M3 | 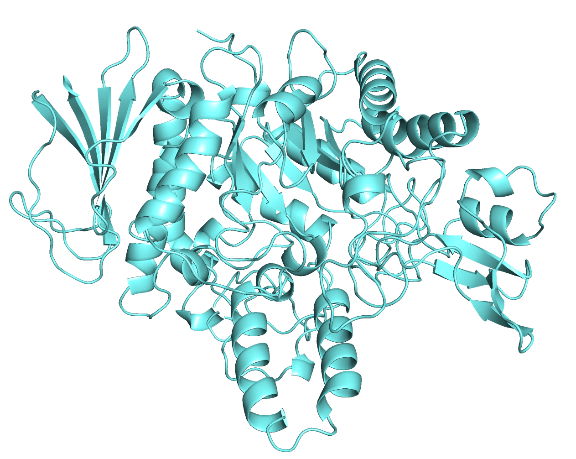 | 92.7 | 95.1 | 0.2 |
| 4 | QSL-M4 | 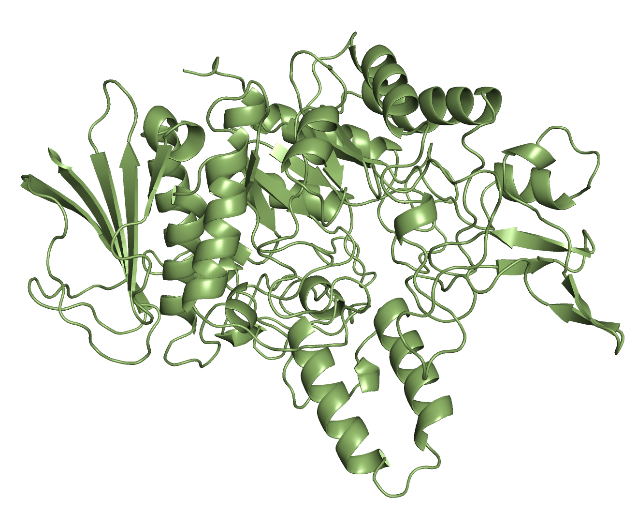 | 91.89 | 95.7 | 0.2 |
| 5 | QSL-M5 | 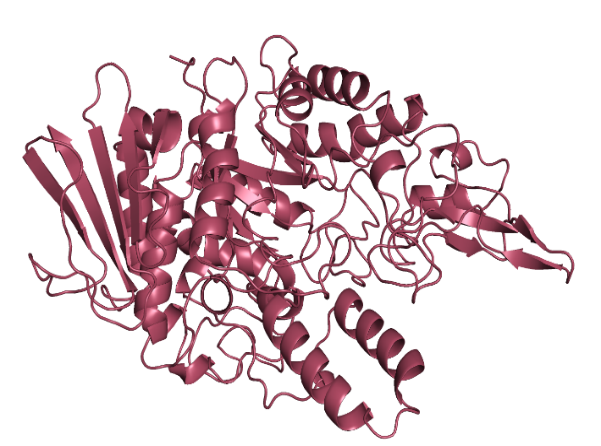 | 91.68 | 95.1 | 0.2 |

**Table S2:** Top 50 FDA-Approved glycomimetic drugs showing lowest Glide Score in XP docking using Schrodinger Glide.

| **Sl. No.** | **Drug** | **Glide score** |
| --- | --- | --- |
| 1 | Acarbose | -11.4717 |
| 2 | Ribostamycin | -10.8977 |
| 3 | Inulin | -9.93883 |
| 4 | Calcium glubionate | -9.53847 |
| 5 | Lincomycin | -9.11977 |
| 6 | Idarubicin | -8.86281 |
| 7 | Lactulose | -8.82533 |
| 8 | Lactose | -8.80864 |
| 9 | Lactitol | -8.73616 |
| 10 | Hydroxyethyl Starch | -8.63156 |
| 11 | Polydatin | -8.59837 |
| 12 | Sorbitol | -8.45395 |
| 13 | Chondroitin sulfate | -8.35498 |
| 14 | Gaxilose | -8.02547 |
| 15 | Decitabine | -7.73085 |
| 16 | Arbutin | -7.57581 |
| 17 | Dapagliflozin | -7.21004 |
| 18 | Migalastat | -7.11438 |
| 19 | Esculin | -7.05611 |
| 20 | 5-O-phosphono-alpha-D-ribofuranosyl diphosphate | -7.02831 |
| 21 | Glucosamine | -7.0279 |
| 22 | Calcium glucoheptonate | -6.98094 |
| 23 | Ticagrelor | -6.87254 |
| 24 | Adenosine | -6.75357 |
| 25 | N-Acetylglucosamine | -6.69087 |
| 26 | Gadobenic acid | -6.66576 |
| 27 | Galactose | -6.55569 |
| 28 | Manganese gluconate | -6.29639 |
| 29 | Gluconic Acid | -6.28932 |
| 30 | Zinc gluconate | -6.19946 |
| 31 | Vidarabine | -6.18147 |
| 32 | Calcium D-saccharate | -6.13861 |
| 33 | Gluconolactone | -6.10851 |
| 34 | Xylose | -6.09635 |
| 35 | Copper gluconate | -6.01235 |
| 36 | Zanamivir | -6.00435 |
| 37 | Calcium gluconate | -5.87312 |
| 38 | Spectinomycin | -5.74324 |
| 39 | Inositol | -5.71329 |
| 40 | Miglustat | -5.59723 |
| 41 | Miglitol | -5.59577 |
| 42 | Trimetrexate | -5.59374 |
| 43 | Calcium glycerophosphate | -5.54396 |
| 44 | Gemcitabine | -5.46608 |
| 45 | Vitamin C | -5.22469 |
| 46 | Novobiocin | -5.21894 |
| 47 | Streptozocin | -5.17313 |
| 48 | Calcium glycerophosphate | -5.02665 |
| 49 | Sodium tartrate | -4.98996 |
| 50 | Sodium glycerophosphate | -4.9584 |

**Table S3:** List of primers used for qRTPCR.

| **S.No.** | **List of Genes** | **Forward Primer** | **Reverse Primer** | **Reference** |
| --- | --- | --- | --- | --- |
|  | *PHR1* | GGTTTGGTTCTGGTTGATGG | AGCAGCAGTTCCTGGACATT | ^49^ |
|  | *PHR2* | CTCCTCCATTTCCAGAACCA | CGTCTGAATCAACCTTGTCG |  |
|  | *SOD1* | TTGAACAAGAATCCGAATCC | AGCCAATGACACCACAAGCAG |  |
|  | *ERG11* | GAAAGAGAACCATTACCAGG | AGGAATCGACGGATCAC | ^34^ |
|  | *CDR* | TGGTGCCATGACTCCTGCTA | CCATCGAGACCAACCCAACA |  |
|  | *MDR* | CCACTGGTGGTGCAAGTGTT | GAACCACAAACAGCACCCAA |  |
|  | *UME6* | AGCACCAAATTCGCCTTATG | AGGTTGAGCTTGCTGCAGTT |  |
|  | *EFG* | CCAGGGTGGTGCTGCTAATG | GGGTGAAGGGTGAACTGAACC |  |
|  | *RBT1* | CTGCCATTCAACCATCTAACTCCTCATAC | GCAGCAAGACCAATAATAGCAGCACCATAAGT |  |
|  | *RAS1* | GGCCATGAGAGAACAATATA | GTCTTTCCATTTCTAAATCAC |  |
